# Lithium Niobate Electro‐Optic Photonic Processor for Variational Quantum Eigensolver

**DOI:** 10.1002/advs.76876

**Published:** 2026-08-18

**Authors:** Jinil Lee, Ui Joon Park, Minho Choi, Hyeong‐Soon Jang, Sunghyun Moon, Hyeon Hwang, Min‐Kyo Seo, Dae‐Hwan Ahn, Sang‐Wook Han, Yong‐Su Kim, Hyounghan Kwon, Hojoong Jung

**Affiliations:** ^1^ Center for Quantum Technology Korea Institute of Science and Technology (KIST) Seoul South Korea; ^2^ Division of Nano & Information Technology KIST School Korea University of Science and Technology Seoul South Korea; ^3^ School of Electrical Engineering Korea University Seoul South Korea; ^4^ Department of Artificial Intelligence Semiconductor Engineering Hanyang University Seoul South Korea; ^5^ Department of Physics Korea Advanced Institute of Science and Technology (KAIST) Daejeon South Korea; ^6^ Division of Quantum Information KIST School Korea University of Science and Technology Seoul South Korea; ^7^ KU‐KIST Graduate School of Converging Science and Technology Korea University Seoul South Korea; ^8^ Department of Physics Kyung Hee University Seoul South Korea

**Keywords:** integrated photonic circuits, lithium niobate on insulator, variational quantum eigensolver

## Abstract

Encoding quantum information in high‐dimensional photonic states as a qudit provides a powerful route to resource‐efficient quantum simulation. Among various integrated photonic platforms, lithium niobate on insulator is particularly attractive because it combines low optical loss, strong optical nonlinearity, and high‐speed electro‐optic modulation. Here, we demonstrate an electro‐optically controlled variational quantum eigensolver (VQE) on an integrated lithium niobate ququart processor. Using ququart encoding in four path modes and electro‐optic modulation, the processor enables reconfigurable high‐fidelity state preparation and projective measurements. To reduce the number of measurement groups, we implement entangled‐basis‐emulating ququart projective measurements that reproduce the measurement‐grouping role of two‐qubit entangled‐basis measurements for fully commuting Pauli operators, without requiring genuine two‐qubit entanglement or entangling gates. Using this approach, we estimate molecular ground‐state energies within the chemical‐accuracy threshold over the measured interatomic‐distance range. We further extend the platform to a chip that integrates a periodically poled lithium niobate photon‐pair source with a ququart photonic processor. These results highlight LNOI photonics as a promising platform for reconfigurable photonic quantum simulation with on‐chip photon‐pair sources.

## Graphical abstract

An integrated lithium‐niobate‐on‐insulator ququart processor implements an electro‐optically controlled photonic variational quantum eigensolver using single photons encoded in four path modes. Bell‐basis‐emulating ququart projective measurements reduce measurement settings while achieving chemical‐accuracy‐level molecular‐energy estimation. A source‐integrated PPLNOI chip further demonstrates VQE operation with on‐chip SPDC photons.

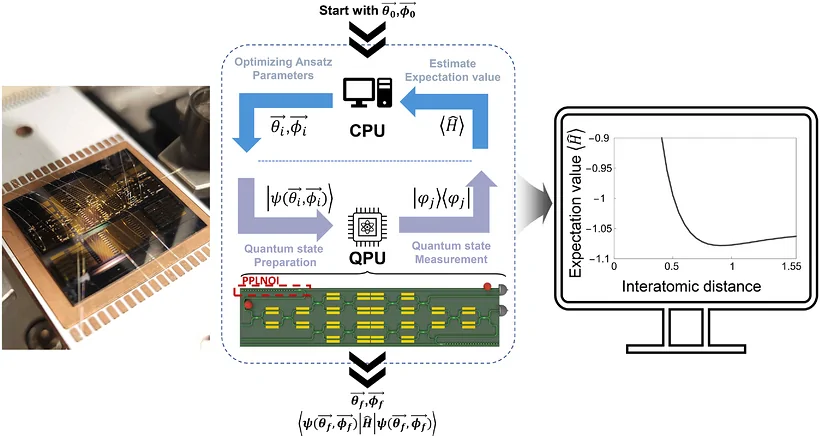

## 1 Introduction

Photons inherently provide multiple high‐dimensional degrees of freedom that can be used to encode quantum states beyond the qubit level [1]. These include path [2, 3, 4, 5, 6, 7], polarization [8, 9], time‐bin [10, 11, 12], frequency‐bin [13, 14, 15], and orbital angular momentum (OAM) [16]. Leveraging such high‐dimensional encodings for qudit‐based quantum information processing offers advantages in quantum simulation, including resource‐efficient Hilbert space representation through enhanced information density. Integrated photonics provides a route to miniaturizing these encodings into reconfigurable on‐chip quantum circuits and has been broadly identified as an enabling platform for scalable quantum technologies [17, 18]. Along this direction, integrated demonstrations based on qudit–qubit mappings, including two‐ququart encodings, have shown programmable multiqubit quantum information processing on chip [19]. However, many widely used platforms, including silicon and silicon nitride photonics, rely on thermally tunable components, which suffer from limited speed and considerable crosstalk [20, 21, 22, 23]. Hybrid integration of electro‐optic (EO) materials has also been explored as an alternative, but this approach inherently increases fabrication complexity [24, 25, 26, 27, 28]. In addition, the limited mode overlap between the photonic mode and the active material often results in reduced modulation efficiency.

Lithium niobate on insulator (LNOI) has emerged as a promising platform for integrated quantum photonic technologies, offering low‐loss waveguides [29], strong second‐order ($\chi^{\left(2\right)}$) nonlinearity, ferroelectric effect, and high‐speed EO modulation [29, 30, 31, 32, 33, 34, 35, 36]. These properties enable a wide range of quantum functionalities such as single‐photon generation [37, 38, 39, 40, 41], quantum interference [42], quantum key distribution [43], frequency conversion [44, 45], and continuous‐variable operations [46] within a single‐material platform, while also providing high‐speed phase modulation for dynamic reconfiguration of photonic circuits [47]. Especially, in continuous‐variable quantum computing systems, periodically poled lithium niobate on insulator (PPLNOI) waveguides play a key role in the generation and measurement of nonclassical states of light, while in modular quantum computing architectures [48, 49], it can serve as a feedforward switching element in refinery circuits, an essential capability for overcoming the probabilistic nature of photonic quantum systems [46]. As a result, LNOI is increasingly regarded as a promising material platform for reconfigurable quantum simulators and processors.

On the other hand, from the algorithmic perspective, hybrid quantum‐classical approaches have recently attracted significant attention. Among the hybrid quantum‐classical algorithms, variational quantum eigensolver (VQE) has gained significant attention in the NISQ (Noisy Intermediate‐Scale Quantum) era for estimating ground‐state energies of molecular systems and quantum materials [50, 51, 52, 53]. In VQE, a parameterized quantum circuit prepares trial states, and the expectation value of a Hamiltonian, which is expressed as a sum of Pauli operators, is measured iteratively. However, as the system size increases, the large number of measurement configurations for quantum simulations has become one of the important experimental challenges [54, 55, 56, 57, 58]. To overcome this challenge, measurement reduction strategies have been proposed in a bulk‐optics VQE system, such as grouping Pauli terms using qubit‐wise commutativity (QWC) or tensor‐product basis (TPB) grouping [53, 59, 60]. While QWC and TPB grouping are limited to partial commutativity, fully commuting Pauli operators can be measured in a common eigenbasis, allowing a common set of projection probabilities to be combined to estimate multiple expectation values [9, 58, 61, 62]. In our ququart encoding, this common‐eigenbasis functionality is implemented through entangled‐basis‐emulating ququart projective measurements, providing a measurement‐grouping route without requiring genuine two‐qubit entanglement or entangling gates.

Despite significant advances in both variational quantum algorithms and LNOI photonic hardware, an experimental demonstration of VQE on an LNOI‐based platform has not yet been achieved. Furthermore, although EO modulation is one of the key advantages of LNOI, its application to dynamic control within variational quantum algorithms has remained unexplored. Here, we report the experimental implementation of the VQE algorithm on a photonic processor based on LNOI, utilizing ququart encoding realized through EO phase control. We demonstrate high‐fidelity preparation and projective measurement of ququart states, employ entangled‐basis‐emulating ququart measurements to reduce the number of required measurement configurations, and reach the chemical‐accuracy threshold over the measured interatomic‐distance range. The present single‐photon, path‐encoded ququart processor serves as a compact EO‐reconfigurable building block for photonic quantum simulation. When combined with integrated photon‐pair generation and additional photonic degrees of freedom, this approach may provide a route toward larger architectures that are not limited to path‐only scaling [1].

## 2 Results

### 2.1 Theory of Variational Quantum Eigensolver and Measurement Grouping

Figure 1 conceptually illustrates the workflow of the chip‐based photonic VQE. Starting from initial ansatz parameters $\left({\vec{\theta }}_{0},{\vec{\phi }}_{0}\right)$, at each iteration i the quantum processing unit (QPU) implements a reconfigurable unitary using tunable beam splitters (θ) and phase shifters (ϕ) to prepare the ansatz state $\left|\psi ({\vec{\theta }}_{i},{\vec{\phi }}_{i})\right\rangle$. The state is then measured in a chosen basis $\left\{\;|\varphi_{j}\right\rangle \left\langle \varphi_{j}|\;\right\}$. The classical processing unit (CPU) reads the measurement outcomes, evaluates $E\left({\vec{\theta }}_{i},{\vec{\phi }}_{i}\right)=\langle \psi \left({\vec{\theta }}_{i},{\vec{\phi }}_{i}\right)|\;\hat{H}\;\left|\psi \left({\vec{\theta }}_{i},{\vec{\phi }}_{i}\right)\right\rangle$, and updates the parameters to $\left({\vec{\theta }}_{i+1},{\vec{\phi }}_{i+1}\right)$ to minimize E. This loop repeats until convergence to $\left({\vec{\theta }}_{f},{\vec{\phi }}_{f}\right)$, at which point the minimized energy $E_{\min}$ approximates the ground state energy $E_{0}$. By repeating the VQE for different interatomic distances R, we reconstruct the potential energy curve $E_{0}\left(R\right)$ as shown in the right panel of Figure 1, identifying its minimum and the equilibrium bond length.

**FIGURE 1:**
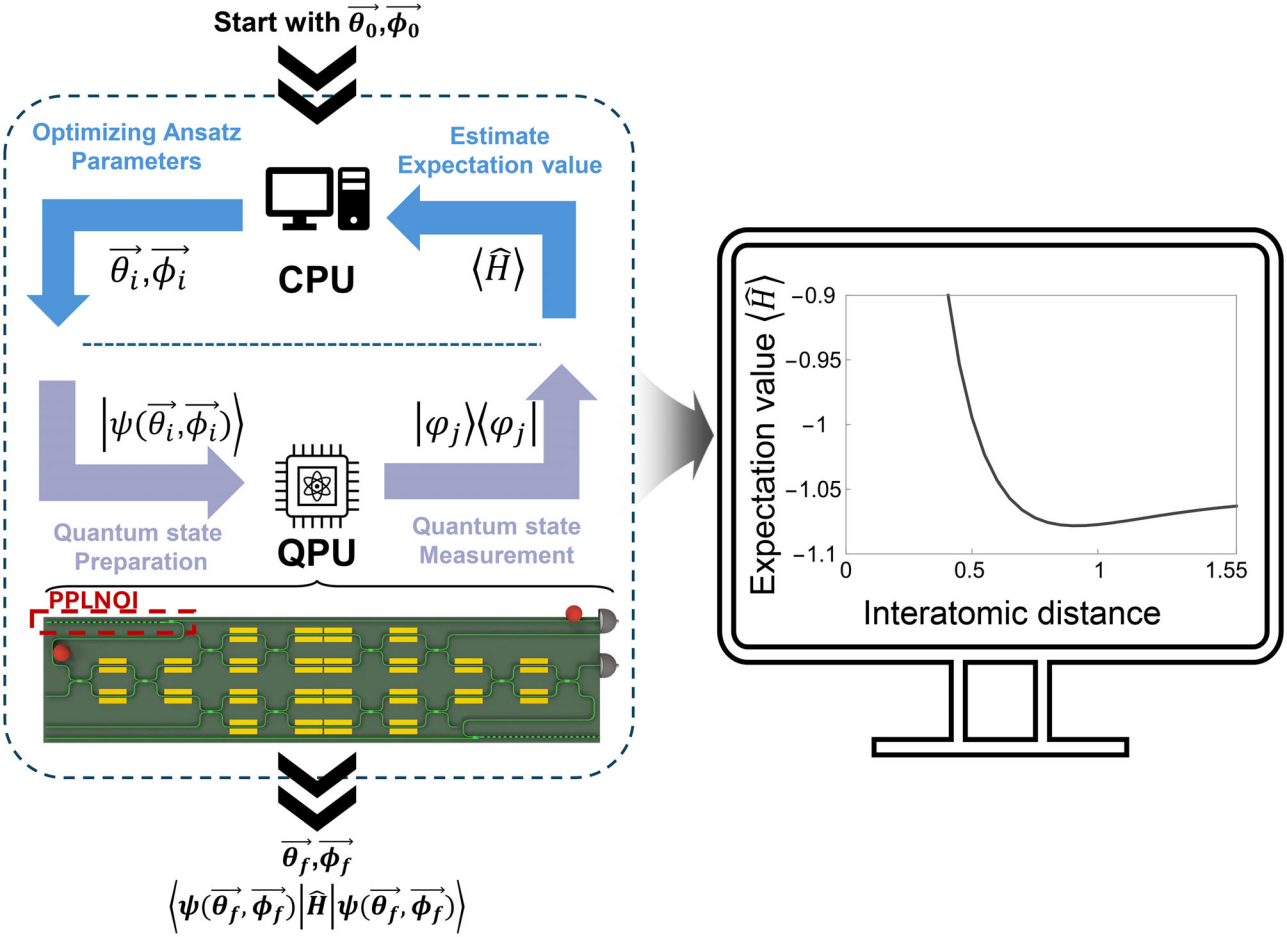
Conceptual schematic of the chip‐based photonic VQE workflow on an integrated LNOI chip. Starting from initial ansatz parameters $\left({\vec{\theta }}_{0},{\vec{\phi }}_{0}\right)$, a classical optimizer (CPU) iteratively calls the quantum processing unit (QPU) to prepare the state $\left|\psi ({\vec{\theta }}_{i},{\vec{\phi }}_{i})\right\rangle$ using an electro‐optic tunable interferometric mesh (chip shown at bottom) and to measure the cost $E\left({\vec{\theta }}_{i},{\vec{\phi }}_{i}\right)=\langle \psi \left({\vec{\theta }}_{i},{\vec{\phi }}_{i}\right)|\hat{H}\left|\psi \left({\vec{\theta }}_{i},{\vec{\phi }}_{i}\right)\right\rangle$, where $\hat{H}=\sum_{k}w_{k}P_{k}$ is decomposed into Pauli strings $P_{k}$. Expectation values $\left\langle P_{k}\right\rangle$ are obtained via sequential measurement. The CPU updates $\left({\vec{\theta }}_{i},{\vec{\phi }}_{i}\right)$ until convergence to $\left({\vec{\theta }}_{f},{\vec{\phi }}_{f}\right)$, yielding the estimate $E_{0}\approx \min_{\vec{\theta },\vec{\phi }}E\left(\vec{\theta },\vec{\phi }\right)$ of the ground state energy. By repeating the VQE for different interatomic distances R, the potential energy curve $E_{0}\left(R\right)$ is reconstructed and its minimum identifies the equilibrium bond length.

To evaluate the energy, we first express the molecular Hamiltonian in second quantization and map it to a multi‐qubit Hamiltonian via the Jordan–Wigner or Bravyi–Kitaev transformation [63]. The resulting Hamiltonian can be written as a weighted sum of Pauli strings.

$$\hat{H}=\sum_{k}\omega_{k}P_{k},\;P_{k}\in {\left\{I,X,Y,Z\right\}}^{⊗n},$$

so that $E=\sum_{k}\omega_{k}\left\langle P_{k}\right\rangle$ is obtained from repeated projective measurements, where $\omega_{k}$ and $P_{k}$ denote the coefficient and the corresponding Pauli string, respectively. To further reduce overhead, we use high‐dimensional projective measurements inspired by two‐qubit entangled‐basis grouping, exploiting fully commuting structures across qubits [9]. In a two‐qubit system, Pauli strings that are fully commuting but not grouped by TPB/QWC can share an entangled common eigenbasis. Measuring in this basis allows one basis setting, or equivalently one common probability distribution, to be used to reconstruct all corresponding expectation values. For example, Bell‐basis measurements in qubit‐based quantum simulators can provide ⟨XX⟩, ⟨YY⟩, and ⟨ZZ⟩ from a common Bell‐basis probability distribution, whereas conventional Pauli‐basis measurements require three distinct groups. Representative entangled common eigenbases and the simultaneously measurable Pauli strings are listed in Table 1.

**TABLE 1:** Representative entangled common eigenbases that group Pauli strings beyond TPB/QWC and the corresponding simultaneously measurable Pauli operators.

Entangled Common Eigenbasis	Measured Pauli Operators
$\frac{1}{\sqrt{2}}\left(|00\right\rangle \pm |11\rangle ),\;\frac{1}{\sqrt{2}}\left(|01\right\rangle \pm \left|10\rangle \right)$	XX,YY,ZZ
$\frac{1}{\sqrt{2}}\left(|0+\right\rangle \pm |1-\rangle ),\;\frac{1}{\sqrt{2}}\left(|0-\right\rangle \pm |1+\rangle )$	XZ,YY,ZX
$\frac{1}{\sqrt{2}}\left(|0i+\right\rangle \pm |1i-\rangle ),\;\frac{1}{\sqrt{2}}\left(|0i-\right\rangle \pm |1i+\rangle )$	XZ,YX,ZY
$\frac{1}{\sqrt{2}}\left(|00\right\rangle \pm i|11\rangle ),\;\frac{1}{\sqrt{2}}\left(|01\right\rangle \pm i|10\rangle )$	XY,YX,ZZ
$\frac{1}{\sqrt{2}}\left(|0+\right\rangle \pm i|1-\rangle ),\;\frac{1}{\sqrt{2}}\left(|0-\right\rangle \pm i|1+\rangle )$	XY,YZ,ZX
$\frac{1}{\sqrt{2}}\left(|0i+\right\rangle \pm i|1i-\rangle ),\;\frac{1}{\sqrt{2}}\left(|0i-\right\rangle \pm i|1i+\rangle )$	XX,YZ,ZY

*Note*: $\left|\pm \right\rangle =\frac{1}{\sqrt{2}}\left(|0\right\rangle \pm |1\rangle ),\;|i\pm \rangle =\frac{1}{\sqrt{2}}\left(|0\right\rangle \pm i|1\rangle )$.

In our ququart implementation, the four path modes of a single photon provide the four‐dimensional basis used to emulate the relevant two‐qubit common‐eigenbasis projectors. These operations are implemented as ququart projective measurements, rather than as genuine nonlocal entangled measurements on two physical qubits. We apply this rule whenever a fully commuting set exists. Such high‐dimensional state preparation and measurement can be realized without CNOT operations, which are known to be challenging in photonic platforms.

### 2.2 Lithium Niobate Quantum Photonic Chip and Experimental Implementation

We use four orthogonal basis states: |0⟩,|1⟩,|2⟩ and |3⟩ based on path modes for ququart encoding. These are mapped to a two‐qubit computational basis as |0⟩≡|00⟩, |1⟩≡|01⟩, |2⟩≡|10⟩, and |3⟩≡|11⟩, so that two‐qubit operations can be implemented within a single four‐dimensional photonic system.

To prepare arbitrary quantum states within the 4D Hilbert space, the chip employs tunable beam splitters and phase shifters. In our implementation, the tunable beam splitter with parameter θ is realized by a Mach–Zehnder interferometer (MZI) consisting of two beam splitters and one phase shifter, while an additional phase shifter with parameter ϕ directly implements the transformation:

$$\left|\psi \right\rangle =\cos\frac{\theta }{2}\;|0\rangle +e^{i\phi }\sin\frac{\theta }{2}\;\left|1\right\rangle .$$

By cascading three such optical modules, an arbitrary ququart state can be prepared as

$$\begin{array}{cc} \left|\psi \right\rangle & =\cos\frac{\theta_{1}}{2}\left(\cos\frac{\theta_{2}}{2}\left|0\right\rangle +e^{i\phi_{2}}\sin\frac{\theta_{2}}{2}\left|1\right\rangle \right)\\ & \;+e^{i\phi_{1}}\sin\frac{\theta_{1}}{2}\left(\cos\frac{\theta_{3}}{2}\left|2\right\rangle +e^{i\phi_{3}}\sin\frac{\theta_{3}}{2}\left|3\right\rangle \right). \end{array}$$

This structured parameterization enables universal state preparation within the 4D space. Due to the symmetry of the circuit in Figure 2a, the same optical circuit can also perform projective measurements on an arbitrary ququart basis. Figure 2a,b shows the schematic of the integrated processor and the photograph of the fabricated device mounted on a Printed Circuit Board (PCB), respectively.

**FIGURE 2:**
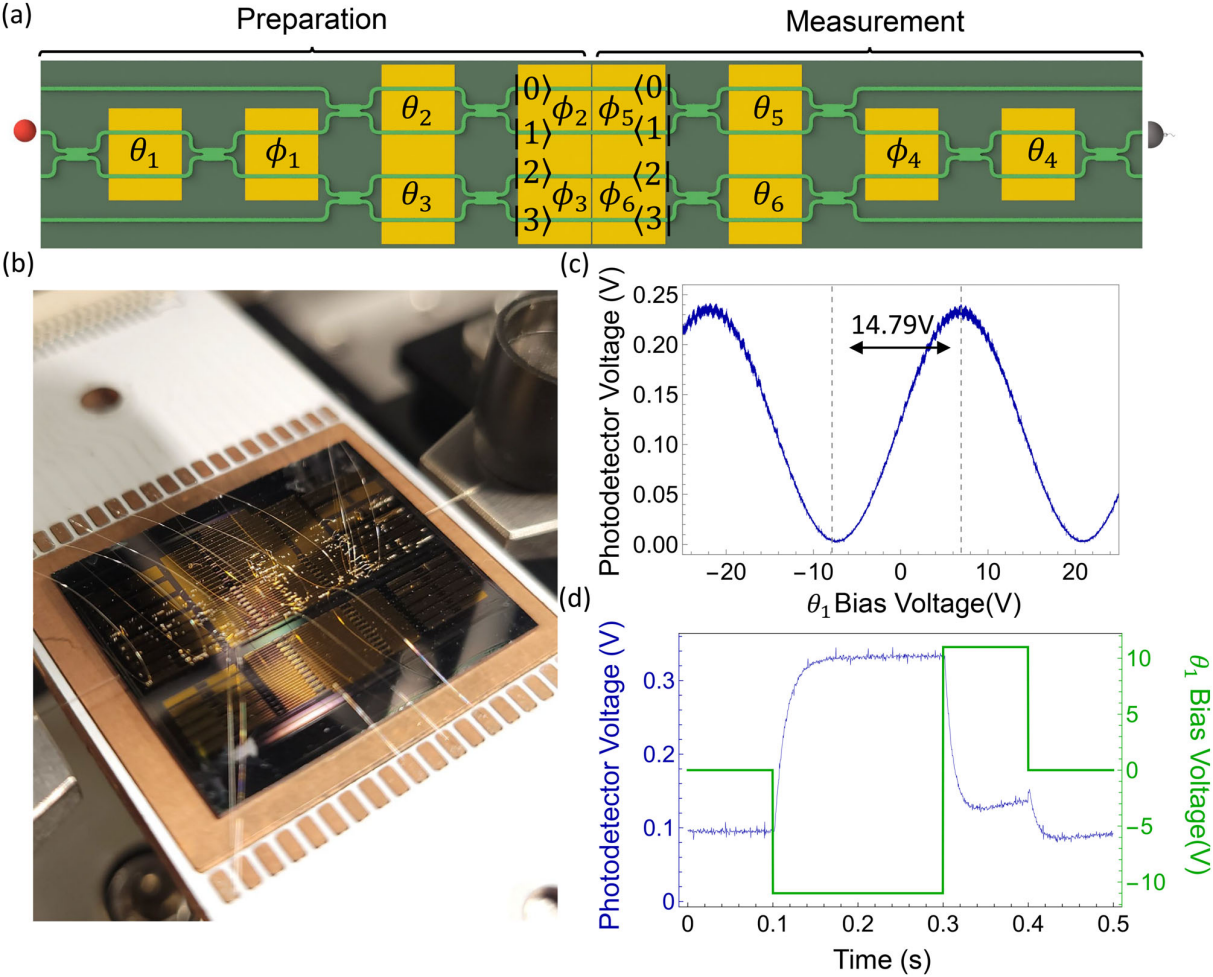
Electro‐optic modulation in an integrated LNOI photonic ququart chip. (a) Schematic layout of the integrated ququart processor, comprising six tunable beam splitters (θ) and six phase shifters (ϕ) for state preparation and measurement. θ controls the power splitting ratio, while ϕ sets the optical phase. (b) Photograph of the fabricated LNOI chip mounted on a PCB and wire‐bonded for electrical drive and control. (c) Photodetector response while sweeping the $\theta_{1}$ bias voltage from −25V to +25V over 1s, showing Mach–Zehnder interference fringes. The extracted half‐wave voltage is $V_{\pi }=14.79\;V$ for an electrode gap of 7μm and an electrode length of 2.45mm. (d) Charge‐balanced drive sequence used during electro‐optic modulation, consisting of 0.1s at 0V, 0.2s at the target bias voltage V, 0.1s at the inverted voltage −V, followed by 0.1s at 0V.

The LNOI processor included twelve on‐chip electro‐optic (EO) elements supporting six tunable beam splitters for θ and six phase shifters for ϕ (see Methods for fabrication details). First, the MMI beam splitters used in the LNOI processor were evaluated using unbalanced MZIs, yielding extinction ratios up to 32.7dB under wavelength sweeps (see Note S1 and Figure S1 for details). To characterize the EO response of the modulators, we applied a linear voltage sweep to the phase shifter in the first MZI $\left(\theta_{1}\right)$ using a continuous‐wave laser at 1560nm‐wavelength. The photodetector signal exhibits sinusoidal fringes, from which we extract a π‐phase voltage $V_{\pi }=14.79\;V$ for a modulator with an electrode length of 2.45mm and a 7μm electrode gap, corresponding to $V_{\pi }L=3.62\;V\;\mathrm{cm}$, as shown in Figure 2c, (see Note S2 and Figure S2 for experimental characterizations of other EO modulators). While no significant DC drift was observed in the long‐term stability experiment (see Note S3 and Figure S3), small offset‐voltage variations motivate the use of a charge‐balanced drive to maintain phase stability during programming and calibration [64]. In our implementation, we employed a sequence of 0.1s at 0V, 0.2s at V, 0.1s at −V, and a final 0.1s at 0V, as shown in Figure 2d, which helps stabilize the programmed phase during repeated operation. This EO modulation enables fast reconfigurable state preparation, shortening the VQE measurement time from several hours to about one hour [8, 9].

### 2.3 On‐Chip Quantum State Tomography of Photonic Ququart and VQE Implementation of Heisenberg Hamiltonian

In this section, we demonstrate on‐chip quantum state tomography (QST) of photonic ququarts and validate the measurement‐grouping advantage of Bell‐basis‐emulating ququart projective measurements using the two‐qubit antiferromagnetic Heisenberg Hamiltonian.

For the characterization of the ququart processor, we employed heralded single photons and a stabilized electro‐optic driving scheme. Heralded single photons at 1560nm were generated in a bulk‐optics system via type‐II spontaneous parametric down‐conversion (SPDC) in a 2mm PPKTP crystal pumped by a 780nm Toptica DLC TA pro laser. One photon served as a herald, and its partner was coupled into the chip through a lensed fiber. EO modulation voltages followed the charge‐balanced drive sequence in Figure 2d. After a brief settling interval, single‐photon counts were integrated over 0.1s, typically yielding ∼3000 counts per projection.

We then characterized our device by performing full QST of on‐chip programmable qudit states. As representative examples, we prepared Bell‐basis‐emulating ququart states, such as $\frac{1}{\sqrt{2}}\left(|0\right\rangle \pm \left|3\rangle \right)$ and $\frac{1}{\sqrt{2}}\left(|1\right\rangle \pm \left|2\rangle \right)$, as shown in Figure 3a. These states were reconstructed with high fidelities and purities. Because the same symmetric ququart circuit is used for both state preparation and the corresponding inverse projection settings, these QST results support the reliability of the Bell‐basis‐emulating measurements. Across all prepared states, we observed an average fidelity of 98.2% for state preparation and an average purity of 98.4% for the projective measurements (See Note S4 and Figure S4 for QST measurement results of various qudit states).

**FIGURE 3:**
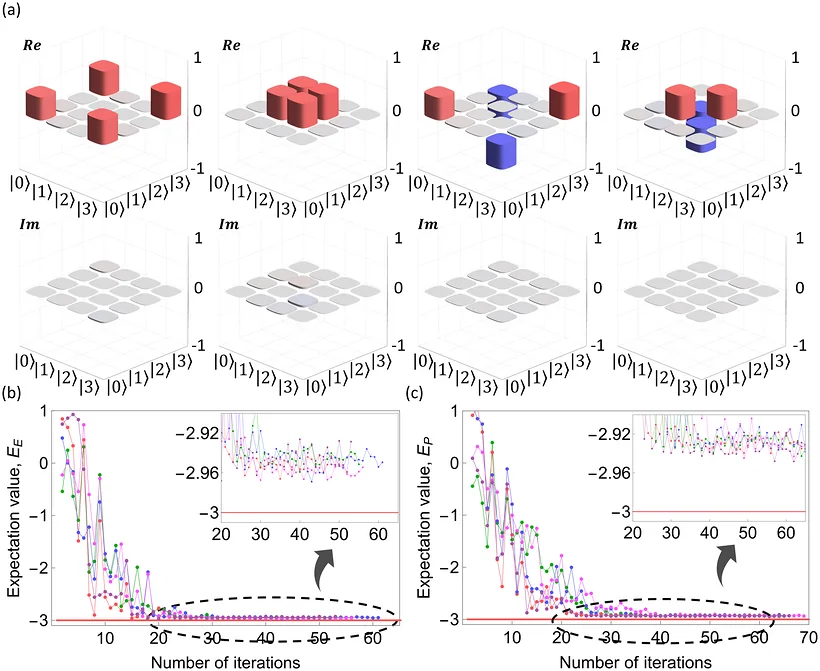
Quantum state tomography of Bell‐basis‐emulating ququart states and application to Heisenberg‐model VQE. (a) Experimental quantum state tomography of four Bell‐basis‐emulating ququart states (left to right). $\frac{1}{\sqrt{2}}\left(\left|0\right\rangle +\left|3\right\rangle \right)$ with F=0.99 and P=0.988. $\frac{1}{\sqrt{2}}\left(\left|1\right\rangle +\left|2\right\rangle \right)$ with F=0.987 and P=0.98. $\frac{1}{\sqrt{2}}\left(\left|0\right\rangle -\left|3\right\rangle \right)$ with F=0.989 and P=0.985. $\frac{1}{\sqrt{2}}\left(\left|1\right\rangle -\left|2\right\rangle \right)$ with F=0.981 and P=0.97. The reconstructed density matrices are shown as real (top row) and imaginary (bottom row) parts. Because the same symmetric ququart circuit is used for both state preparation and the corresponding inverse projection settings, these QST results provide an operational check of the Bell‐basis‐emulating measurements. (b) VQE results for the two‐qubit antiferromagnetic Heisenberg Hamiltonian $\hat{H}=XX+YY+ZZ$ implemented on an integrated LNOI ququart chip, with expectation values estimated using Bell‐basis‐emulating ququart measurements ($E_{E}$). (c) Corresponding VQE results obtained with Pauli‐basis measurements ($E_{P}$). In both cases, each curve represents one of five independent VQE runs. The insets compare ($E_{E}$) and ($E_{P}$), showing that ($E_{E}$) converges more rapidly and remains closer to the theoretical eigenvalue due to the reduced number of required circuit reconfigurations (four versus twelve).

Next, we turned to the Heisenberg model, which provides a well‐known Hamiltonian for exploring critical points and phase transitions in magnetic systems. In the two‐qubit antiferromagnetic case we take $\hat{H}=XX+YY+ZZ$, where the Bell basis forms a common eigenbasis of $\hat{H}$. Consequently, Bell‐basis‐emulating ququart projections are sufficient to reconstruct ⟨XX⟩, ⟨YY⟩, and ⟨ZZ⟩, and thus $\left\langle \hat{H}\right\rangle$. The set {XX,YY,ZZ} is fully commuting yet not QWC, which makes this model one of the ideal examples for the measurement‐grouping strategy. Indeed, the QST results in Figure 3a show that the processor can reliably access the corresponding Bell‐basis‐emulating ququart settings with high fidelity and purity, making the Heisenberg Hamiltonian a natural testbed for validating our Bell‐basis‐emulating measurement approach. All three expectation values are obtained from a single ququart basis group with four orthogonal projections, whereas conventional Pauli‐basis estimation requires three separate groups consisting of twelve total projections.

Figure 3b,c shows VQE experiments on the two‐qubit antiferromagnetic Heisenberg Hamiltonian using two measurement strategies. The red line marks the exact ground state energy (−3). Figure 3b shows the ground energy expectation value, $E_{E}$, obtained with Bell‐basis‐emulating ququart measurements. Each iteration uses one ququart basis group with four orthogonal projections. Figure 3c shows the ground energy expectation value, $E_{P}$, with conventional Pauli‐basis measurements. Each iteration requires three distinct groups with four projections per group, for a total of twelve projections. Five independent runs are plotted in each case.

In both methods, the energy approaches the ground state value as the optimization proceeds. The dashed ellipse marks the saturated iteration region. In the zoom‐in insets, the traces with Bell‐basis‐emulating ququart measurements lie slightly closer to the exact ground state energy (−3) and reach it in fewer iterations than the traces with Pauli basis measurements. This trend is consistent with the common‐eigenbasis grouping principle for fully commuting Pauli strings [62]. Because each projection setting was measured sequentially in our photon‐counting setup, the benefit should be understood as a reduction in the required measurement settings and circuit reconfigurations, rather than as simultaneous multi‐output readout. This reduction lowers reconfiguration overhead and mitigates errors introduced when switching between measurement settings.

### 2.4 Chemical‐Accuracy‐Level VQE with Reduced Measurement Settings

We implemented VQE for the He–$H^{+}$ molecule on our integrated LNOI photonic ququart chip with an emphasis on reducing the number of measurement groups using Bell‐basis‐emulating ququart measurements. After mapping the molecular Hamiltonian to two qubits, the resulting Pauli‐string Hamiltonian contains nine terms [51]:

$$\begin{array}{cc} \hat{H} & =w_{\mathrm{II}}\;II+w_{\mathrm{IZ}}\;IZ+w_{\mathrm{ZI}}\;ZI+w_{\mathrm{ZZ}}\;ZZ+w_{\mathrm{IX}}\;IX+w_{\mathrm{ZX}}\;ZX\\ & \;+w_{\mathrm{XI}}\;XI+w_{\mathrm{XZ}}\;XZ+w_{\mathrm{XX}}\;XX. \end{array}$$

Grouping by qubit‐wise commutativity (QWC) yields four measurement groups, each resolved with four orthogonal projections, so evaluating $\hat{H}$ requires sixteen configurations per VQE iteration. To reduce this cost, we exploit full commutativity and partition the nine terms into three groups. The set {XX,ZZ,II} is measured in a single Bell‐basis‐emulating ququart basis group, while {XI,XZ,IZ} and {IX,ZI,ZX} are measured in the Pauli basis. This reduces the number of total measurement configurations from sixteen to twelve.

The overall performance of the VQE experiment is summarized in Figure 4. At the equilibrium bond distance R=0.9Å, which corresponds to the minimum of the potential energy curve, Figure 4a shows the bias voltages for θ and ϕ during optimization, and Figure 4b shows the convergence of the measured energy (blue points) toward the theoretical ground state value (red line). At this distance, we estimate the ground‐state energy by averaging the five lowest measured energy values, with uncertainties obtained by error propagation from detected single photon counts. Using this five‐lowest‐energy average, we obtain −1.09034±0.0023Ha, in agreement with the theoretical value −1.09031Ha and within the chemical‐accuracy threshold of 1.6mHa. The sensitivity of the chemical‐accuracy conclusion to the number of lowest‐energy points included in the average is analyzed in Supplementary Note 5.

**FIGURE 4:**
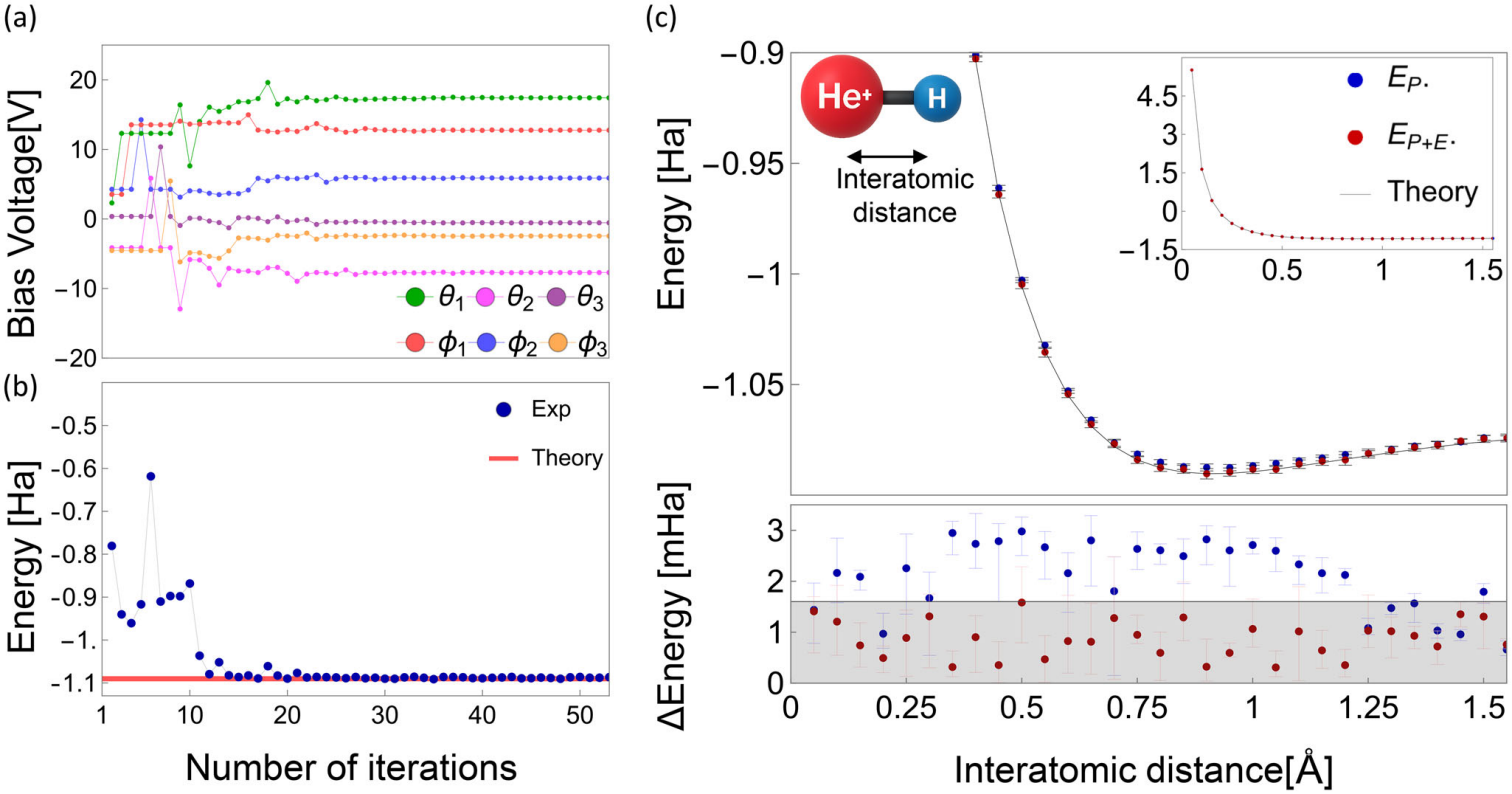
Photonic VQE for the He–H 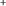 ground state energy. Results at an interatomic distance of R=0.9Å, where both Bell‐basis‐emulating ququart and Pauli‐basis measurements were performed during VQE. (a) Bias voltages applied to the θ (tunable beam splitters) and ϕ (phase shifters) showing how the circuit parameters evolve under classical optimization toward the energy minimum. (b) Convergence of the experimentally measured energy (blue points) to the theoretical ground state value (red line) as the iteration number increases. (c) Potential energy curve $E_{0}\left(R\right)$ obtained via VQE for He–$H^{+}$ as a function of interatomic distance R, measured on our integrated photonic ququart chip. Experimental estimates are shown as markers: $E_{P}$ (blue, grouped Pauli‐basis measurements) and $E_{P+E}$ (red, fully commuting Pauli groups measured with a combination of Bell‐basis‐emulating ququart and Pauli‐basis measurements), alongside the theoretical curve (solid line). The lower panel shows the deviation ΔE of the experimental results from theory. The red dots are all in the shaded region indicating chemical accuracy (≤1.6mHa).

We then varied R from 0.05 to 1.55Å to reconstruct the potential energy curve, as shown in Figure 4c. Results using Pauli‐only measurements are denoted $E_{P}$ (blue), and results using Pauli plus Bell‐basis‐emulating ququart measurements are denoted $E_{P+E}$ (red). Using the same average of the five lowest energy values, the $E_{P+E}$ data show consistently smaller deviations from theory, with all measured distances falling within the chemical accuracy threshold of 1.6mHa. In the lower panel, the error bars indicate the range (minimum to maximum) among the five values used to compute the average at each distance. This trend of reduced errors with Bell‐basis‐emulating ququart measurements is consistent with the behavior observed in Figure 3b,c, and can be attributed to the reduction in measurement groups, which lowers reconfiguration overhead and reduces calibration drift and switching‐induced noise.

### 2.5 Photonic VQEs with an Integrated Quantum Light Source

Integrating quantum light sources with reconfigurable photonic processors is a critical step toward scalable photonic quantum systems with on‐chip sources. PPLNOI provides a natural route for this integration because periodic poling enables on‐chip $\chi^{\left(2\right)}$ photon‐pair generation on the same material platform used for EO‐reconfigurable processing. In particular, moving from bulk or off‐chip photon sources to chips that integrate the source and processor can reduce alignment complexity, improve mechanical stability, and simplify the deployment of variational quantum algorithms on a chip‐scale platform.

To this end, we fabricated a second chip integrating a PPLNOI source waveguide with the ququart processor, as illustrated in Figure 5a. All SHG/SPDC characterization, PPLNOI‐source QST, and source‐integrated VQE measurements were performed on the same source‐integrated PPLNOI processor shown in Figure 5a, not on a separate test structure. In Figure 5b, the SHG peak observed at a fundamental wavelength of 1575.23nm is consistent with quasi‐phase‐matched operation of the designed PPLNOI source. To further characterize this process, the SPDC spectra were acquired using a near‐visible tunable laser, as shown in Figure S6. Figure S7 shows a second‐harmonic generation (SHG) microscopy image of the poled section. Specifically, these SPDC count sweeps then showed a filtered coincidence response near the operating pump wavelength with an angle‐tuned 1580nm, 12nm‐bandwidth band‐pass filter, as shown in Figure S6. The QST and VQE measurements used a pump wavelength of 788.25nm, corresponding to a nominal degenerate SPDC wavelength of 1576.5nm. The generated photon pairs were separated by an on‐chip MMI beam splitter, with one photon used for heralding at D1 and the other injected into the ququart circuit and detected at D2.

**FIGURE 5:**
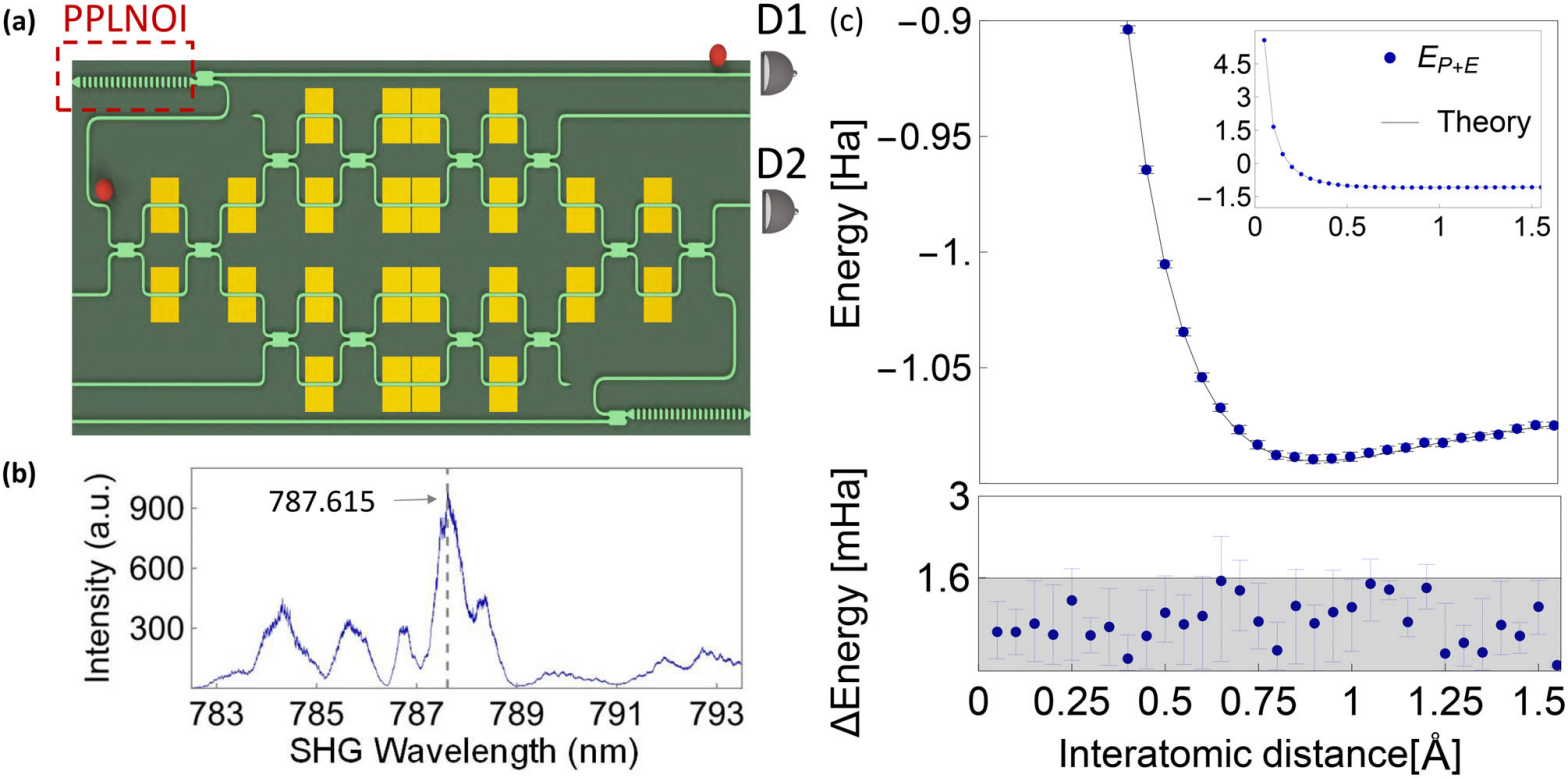
VQE with heralded single photons from the integrated PPLNOI source. (a) Schematic of the periodically poled lithium niobate section integrated on the LNOI chip. Type‐0 SPDC generates a photon pair. One photon is routed to D1 for heralding, while the other is injected into the ququart circuit and detected at D2. (b) SHG spectrum measured in the PPLNOI section while sweeping the fundamental wavelength. The SHG peak at a fundamental wavelength of 1575.23nm is consistent with quasi‐phase‐matched operation of the designed PPLNOI source and corresponds, by reciprocity, to degenerate type‐0 SPDC when pumped at ∼787.615nm. (c) Potential‐energy curve $E_{0}\left(R\right)$ obtained via VQE for He–$H^{+}$ using heralded single photons from the integrated PPLNOI source. The upper panel shows the average of the five lowest measured energies at each interatomic distance together with the theoretical curve. The lower panel shows the corresponding deviation from theory, with error bars indicating the range among the five values used in the average. The shaded region indicates chemical accuracy (≤1.6mHa).

Using the integrated PPLNOI source, we performed QST of ququart states, summarized in Note S6 and Figure S10, and repeated the He–$H^{+}$ VQE with the reduced‐measurement strategy. Figure 5c shows the resulting potential‐energy reconstruction together with the theoretical prediction. The minimum of the reconstructed curve was obtained at R=0.90Å, where the average of the five lowest measured energies was E=−1.0895±0.0019Ha. The lowest‐five average remains within chemical accuracy over the measured range. Measurement conditions, timing protocol, voltage sweeps, and bias‐on transient characterizations are provided in Methods, Note S6, and Figures S8 and S9. Overall, these results demonstrate integration of a PPLNOI photon‐pair source with an LNOI ququart processor and show that the integrated PPLNOI ququart processor supports both QST and VQE measurements with on‐chip SPDC photons.

## 3 Discussion

In summary, we have demonstrated VQE on an LNOI photonic ququart processor and shown several device‐level capabilities relevant to integrated quantum photonics. These include high‐speed electro‐optic control within a VQE loop, reduced measurement settings enabled by entangled‐basis‐emulating ququart projective measurements, and molecular‐energy estimation assessed using the five‐lowest‐energy estimator and compared with the chemical‐accuracy threshold. We also extended the platform by integrating a PPLNOI section as an on‐chip quantum light source, demonstrating photon‐pair generation and reconfigurable processing on the same material platform. Table 2 summarizes the comparison between our platform and previous photonic VQE demonstrations in terms of phase‐shifter technology, integrated photon sources, and chemical accuracy.

**TABLE 2:** Summary of VQE experiments on photonic devices.

Platform	Phase shifter	Integrated photon source	Chemical accuracy
Silicon (2014) [51]	Thermo‐optic	No (off‐chip SPDC)	Yes (corrected for experimental errors)
Silicon (2023) [3]	Thermo‐optic	Yes (on‐chip SFWM)	Yes (bias‐corrected)
Silicon (2025) [2]	Thermo‐optic	Yes (on‐chip SFWM)	Near (∼0.003 Ha)
Silica (2023) [65]	Thermo‐optic	No (off‐chip SPDC)	—
Silica (2024) [66]	Thermo‐optic	No (off‐chip SPDC)	—
SiN (2024) [67]	Thermo‐optic	No (external QD feeding PIC)	Yes (machine‐learned transpilation)
LNOI (This work)	Electro‐optic	on‐chip SPDC	Yes (lowest‐five average)

The present single‐photon, path‐encoded ququart demonstration may have inherent limits if scaled via path alone. That is because the number of required spatial modes increases exponentially with the number of encoded qubits, rapidly increasing the chip footprint and accumulating propagation, bending, and splitter losses. Instead, it serves as a compact building block that demonstrates electro‐optically reconfigurable ququart control together with on‐chip photon‐pair generation. A more practical route is to combine high‐dimensional encoding with other photonic degrees of freedom, such as frequency‐bin modes, time‐bin modes, polarization, or continuous‐variable modes, while using LNOI electro‐optic control for fast reconfiguration. In this route, source integration can be advantageous because entangled qudit photon pairs generated in PPLNOI sections can effectively support entanglement across these diverse degrees of freedom that path‐only single‐photon encoding lacks [42]. In particular, integrating multiple PPLNOI sections on the same chip is expected to provide a route to high‐dimensional entangled photon‐pair generation and to enable access to entangled two‐photon qudit states. Such hybrid‐encoding architectures with integrated photon‐pair sources provide a route toward larger implementations of variational quantum algorithms.

Fast reconfigurable processors are important for photonic quantum systems, and EO modulation is well suited to this role. In quantum simulation, for example, the number of measurements in VQEs grows rapidly with system size, making rapid circuit updates beneficial for reducing experimental overhead. More broadly, photonic quantum systems rely on multiplexing and feedforward, as illustrated in continuous‐variable modular architectures where LNOI binary‐tree switches have served as refinery elements [46]. Building on this architectural concept, our device shares a fundamentally similar design with these refinery circuits, while implementing a large array of integrated phase modulators designed to continuously control both optical phases and splitting ratios rather than acting solely as binary switches. This versatile control scheme provides a highly functional, programmable hardware foundation applicable to a wide range of advanced photonic quantum technologies.

Taken together, these results show that a lithium niobate EO ququart processor can produce molecular‐energy estimates assessed using the five‐lowest‐energy estimator and can be combined with an integrated PPLNOI photon‐pair source. The present path‐encoded device is a compact experimental building block rather than a complete large‐scale architecture, but the combination of EO programmability, $\chi^{\left(2\right)}$ photon‐pair generation, and compatibility with hybrid encodings makes LNOI a promising material platform for future quantum photonic systems.

## 4 Methods

### 4.1 Device Fabrication

The photonic ququart chip was fabricated using 300 nm‐thick x‐cut MgO‐doped LN film on 4.77 μm‐thick silicon dioxide on 525 μm‐thick silicon substrate from NanoLN. Rib waveguides with a width of 1 μm were patterned using electron‐beam lithography (EBL) with Ma‐N 2410 negative resist. LN patterns were etched using inductively coupled plasma reactive ion etching (ICP‐RIE) with Ar gas to a depth of 240 nm, leaving 60 nm‐thick slab.

After etching, the e‐beam resist residue and redeposition were removed by KOH cleaning process. A 2 μm‐thick ${\mathrm{SiO}}_{2}$ cladding layer was deposited using plasma‐enhanced chemical vapor deposition (PECVD), followed by thermal annealing at 600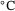 for 1 h.

For electro‐optic modulation, metal electrodes were fabricated on top of the cladding. First, DNR‐L300‐21.8cP photoresist was spin‐coated and patterned via maskless lithography. After etching 1.95 μm of the ${\mathrm{SiO}}_{2}$ cladding using RIE, the remaining oxide was removed using buffered oxide etch (BOE) for 2 min. Without removing the photoresist, 10 nm‐thick chromium and 100 nm‐thick gold were deposited using electron‐beam evaporation. The lift‐off process completed the formation of the electrode layer.

We polished each facet of the chip using an Allied MultiPrep system with the following sequential polishing steps: 30 and 3 μm diamond lapping films, Tech‐Cloth polishing with 3 μm diamond suspension, and final cleaning using a soap‐and‐water solution.

All twelve electro‐optic modulators are driven by a National Instruments data‐acquisition (DAQ) unit with twelve synchronized analog voltage outputs. Because the required voltage swing exceeds the native DAQ range, each channel feeds a high‐voltage amplifier that provides a ±25V output to the electrodes.

A second device integrating a periodically poled LNOI waveguide together with the ququart processor was fabricated on x‐cut LNOI without MgO doping. The PPLNOI source section was designed with a 3.2μm poling period. The source‐waveguide width was swept from 2.30 to 2.40μm, with 2.36μm used as the target width. The approximate fiber‐to‐fiber insertion loss was ∼6dB for the MgO‐doped LNOI processor and ∼13dB for the PPLNOI integrated device. In this device, slow EO phase‐offset variation during sequential measurements was more noticeable than in the MgO‐doped LNOI processor. Motivated by previous reports on DC‐stable thin‐film lithium tantalate electro‐optic devices [68], we applied post‐fabrication oxygen annealing at $300^{\circ }C$ for 3 h in $O_{2}$ atmosphere as a stabilization step. We do not treat this process as a universal or complete suppression of DC‐related EO phase‐offset variation. Slow relaxation and drift in thin‐film lithium niobate electro‐optic modulators have also been reported previously [69].

To maintain stable operation over measurement time, automatic fiber‐to‐chip coupling alignment using a piezo stage was performed every few VQE runs to maximize the detected count rate. During the PPLNOI‐source measurements, 350μW of off‐chip pump power was coupled into the PPLNOI waveguide using a lensed fiber, and the device temperature was set to $20^{\circ }C$, slightly below room temperature, to help mitigate DC drift during the measurements. For each PPLNOI projection, the EO electrodes were held at 0V for 0.8s before the target voltage was applied. After a 0.3s delay for timing synchronization, photon counts were integrated for 0.1s to reduce the influence of slow EO phase‐offset variation during acquisition. The electrodes were then biased with the opposite polarity for 0.4s before the next setting. Estimated accidental coincidences were subtracted before reconstructing projection probabilities. COBYQA was used as the classical optimizer.

## Conflicts of Interest

The authors declare no conflicts of interest.

## Supporting information


**Supporting File**: advs76876‐sup‐0001‐SuppMat.pdf.

## Data Availability

The data that support the findings of this study are available from the corresponding author upon request.

## References

[1] M. Erhard , M. Krenn , and A. Zeilinger , “Advances in High‐Dimensional Quantum Entanglement,” Nature Reviews Physics 2, no. 7 (2020): 365–381.

[2] A. Baldazzi , M. Sanna , M. Borghi , S. Azzini , and L. Pavesi , “Four‐Qubit Variational Algorithms in Silicon Photonics with Integrated Entangled Photon Sources,” npj Quantum Information 11, no. 1 (2025): 107.

[3] Y. Wang , S. Xue , Y. Wang , et al., “Experimental Quantum Natural Gradient Optimization in Photonics,” Optics Letters 48, no. 14 (2023): 3745–3748. 10.1364/OL.494560 37450740

[4] J.‐M. Lee , J. Park , J. Bang , et al., “Quantum States Generation and Manipulation in a Programmable Silicon‐Photonic Four‐Qubit System with High‐Fidelity and Purity,” APL Photonics 9, no. 7 (2024): 076110.

[5] N. C. Harris , D. Bunandar , M. Pant , et al., “Large‐Scale Quantum Photonic Circuits in Silicon,” Nanophotonics 5, no. 3 (2016): 456–468.

[6] X. Qiang , X. Zhou , J. Wang , et al., “Large‐Scale Silicon Quantum Photonics Implementing Arbitrary Two‐Qubit Processing,” Nature Photonics 12, no. 9 (2018): 534–539.

[7] Y. Yu , Y. Chi , C. Zhai , J. Huang , Q. Gong , and J. Wang , “Programmable Silicon‐Photonic Quantum Simulator Based on a Linear Combination of Unitaries,” Photonics Research 12, no. 8 (2024): 1760–1767.

[8] D. Lee , J. Lee , S. Hong , et al., “Error‐Mitigated Photonic Variational Quantum Eigensolver Using a Single‐Photon Ququart,” Optica 9, no. 1 (2022): 88–96, 10.1364/OPTICA.441163.

[9] J. Lee , W. Song , D. Lee , et al., “Photonic Variational Quantum Eigensolver Using Entanglement Measurements,” Quantum Science and Technology 9, no. 4 (2024): 045028.

[10] I. Marcikic , H. de Riedmatten , W. Tittel , V. Scarani , H. Zbinden , and N. Gisin , “Time‐Bin Entangled Qubits for Quantum Communication Created by Femtosecond Pulses,” Physical Review A 66, no. 6 (2002): 062308.

[11] T. Inagaki , N. Matsuda , O. Tadanaga , M. Asobe , and H. Takesue , “Entanglement Distribution over 300 km of Fiber,” Optics Express 21, no. 20 (2013): 23241–23249. 10.1364/OE.21.023241 24104238

[12] J. Yoo , Y. Choi , Y.‐W. Cho , et al., “Experimental Preparation and Characterization of Four‐Dimensional Quantum States Using Polarization and Time‐Bin Modes of a Single Photon,” Optics Communications 419 (2018): 30–35.

[13] C. Reimer , M. Kues , P. Roztocki , et al., “Generation of Multiphoton Entangled Quantum States by Means of Integrated Frequency Combs,” Science 351, no. 6278 (2016): 1176–1180. 10.1126/science.aad8532 26965623

[14] M. Kues , C. Reimer , P. Roztocki , et al., “On‐Chip Generation of High‐Dimensional Entangled Quantum States and Their Coherent Control,” Nature 546, no. 7660 (2017): 622–626. 10.1038/nature22986 28658228

[15] M. Clementi , F. A. Sabattoli , M. Borghi , et al., “Programmable Frequency‐Bin Quantum States in a Nano‐Engineered Silicon Device,” Nature Communications 14, no. 1 (2023): 176. 10.1038/s41467-022-35773-6 PMC9837142 36635283

[16] B. Kim , K.‐M. Hu , M.‐H. Sohn , et al., “Qudit‐Based Variational Quantum Eigensolver Using Photonic Orbital Angular Momentum States,” Science Advances 10, no. 43 (2024): eado3472. 10.1126/sciadv.ado3472 PMC11498208 39441921

[17] J. Wang , F. Sciarrino , A. Laing , and M. G. Thompson , “Integrated Photonic Quantum Technologies,” Nature Photonics 14, no. 5 (2020): 273–284.

[18] E. Pelucchi , G. Fagas , I. Aharonovich , et al., “The Potential and Global Outlook of Integrated Photonics for Quantum Technologies,” Nature Reviews Physics 4, no. 3 (2022): 194–208.

[19] J. Huang , X. Li , X. Chen , et al., “Demonstration of Hypergraph‐State Quantum Information Processing,” Nature Communications 15 (2024): 2601, 10.1038/s41467-024-46830-7. PMC10960808 38521765

[20] J. Carolan , C. Harrold , C. Sparrow , et al., “Universal Linear Optics,” Science 349, no. 6249 (2015): 711–716. 10.1126/science.aab3642 26160375

[21] C. Sparrow , E. Martín‐López , N. Maraviglia , et al., “Simulating the Vibrational Quantum Dynamics of Molecules Using Photonics,” Nature 557, no. 7707 (2018): 660–667. 10.1038/s41586-018-0152-9 29849155

[22] J. Wang , S. Paesani , Y. Ding , et al., “Multidimensional Quantum Entanglement with Large‐Scale Integrated Optics,” Science 360, no. 6386 (2018): 285–291. 10.1126/science.aar7053 29519918

[23] J. M. Arrazola , V. Bergholm , K. Brádler , et al., “Quantum Circuits with Many Photons on a Programmable Nanophotonic Chip,” Nature 591, no. 7848 (2021): 54–60. 10.1038/s41586-021-03202-1 PMC11008968 33658692

[24] L. Chen , Q. Xu , M. G. Wood , and R. M. Reano , “Hybrid Silicon and Lithium Niobate Electro‐Optical Ring Modulator,” Optica 1, no. 2 (2014): 112–118.

[25] M. Churaev , R. N. Wang , A. Riedhauser , et al., “A Heterogeneously Integrated Lithium Niobate‐on‐Silicon Nitride Photonic Platform,” Nature Communications 14, no. 1 (2023): 3499. 10.1038/s41467-023-39047-7 PMC10264395 37311746

[26] M. Niels , T. Vanackere , E. Vissers , et al., “A High‐Speed Heterogeneous Lithium Tantalate Silicon Photonics Platform,” *arXiv* (2025): arXiv:2503.10557.

[27] J. Cai , A. Kotz , H. Larocque , et al., “Heterogeneously Integrated Lithium Tantalate‐on‐Silicon Nitride Modulators for High‐Speed Communications,” *arXiv* (2025): arXiv:2508.06265. 10.1038/s41467-026-69769-3 PMC13066381 41764154

[28] “A Manufacturable Platform for Photonic Quantum Computing,” Nature 641, no. 8064 (2025): 876–883. 10.1038/s41586-025-08820-7 PMC12095036 40010377

[29] B. Desiatov , A. Shams‐Ansari , M. Zhang , C. Wang , and M. Lončar , “Ultra‐Low‐Loss Integrated Visible Photonics Using Thin‐Film Lithium Niobate,” Optica 6, no. 3 (2019): 380–384.

[30] D. Zhu , L. Shao , M. Yu , et al., “Integrated Photonics on Thin‐Film Lithium Niobate,” Advances in Optics and Photonics 13, no. 2 (2021): 242–352.

[31] Y. Qi and Y. Li , “Integrated Lithium Niobate Photonics,” Nanophotonics 9, no. 6 (2020): 1287–1320.

[32] K. Luke , P. Kharel , C. Reimer , L. He , M. Loncar , and M. Zhang , “Wafer‐Scale Low‐Loss Lithium Niobate Photonic Integrated Circuits,” Optics Express 28, no. 17 (2020): 24452–24458. 10.1364/OE.401959 32906986

[33] A. Boes , L. Chang , C. Langrock , et al., “Lithium Niobate Photonics: Unlocking the Electromagnetic Spectrum,” Science 379, no. 6627 (2023): eabj4396. 10.1126/science.abj4396 36603073

[34] S. Saravi , T. Pertsch , and F. Setzpfandt , “Lithium Niobate on Insulator: An Emerging Platform for Integrated Quantum Photonics,” Advanced Optical Materials 9, no. 22 (2021): 2100789.

[35] M. G. Vazimali and S. Fathpour , “Applications of Thin‐Film Lithium Niobate in Nonlinear Integrated Photonics,” Advanced Photonics 4, no. 3 (2022): 034001–034001.

[36] H.‐S. Jang , H. Heo , S. Kim , et al., “Fabrication of a 3D Mode Size Converter for Efficient Edge Coupling in Photonic Integrated Circuits,” Optics Express 33, no. 4 (2025): 6909–6917. 10.1364/OE.541701 40798425

[37] J. Zhao , C. Ma , M. Rüsing , and S. Mookherjea , “High Quality Entangled Photon Pair Generation in Periodically Poled Thin‐Film Lithium Niobate Waveguides,” Physical Review Letters 124, no. 16 (2020): 163603. 10.1103/PhysRevLett.124.163603 32383916

[38] J. Lu , A. Al Sayem , Z. Gong , J. B. Surya , C.‐L. Zou , and H. X. Tang , “Ultralow‐Threshold Thin‐Film Lithium Niobate Optical Parametric Oscillator,” Optica 8, no. 4 (2021): 539–544.

[39] K. Kwon , H. Heo , D. Lee , et al., “Photon‐Pair Generation Using Inverse‐Designed Thin‐Film Lithium Niobate Mode Converters,” APL Photonics 9, no. 5 (2024): 056108.

[40] C. Kim , M. Bae , M. Choi , et al., “Freeform Thin‐Film Lithium Niobate Mode Converter for Photon‐Pair Generation,” Nanophotonics 14, no. 11 (2025): 1949–1960. 10.1515/nanoph-2024-0515 PMC12133314 40470073

[41] C. Kim , H. Kim , M. Choi , et al., “Integrated Bright Source of Polarization‐Entangled Photons Using Lithium Niobate Photonic Chips,” *arXiv* (2025): arXiv:2506.23625.

[42] S. Moon , J. Lee , J. Lee , et al., “Broadband Thin‐Film Lithium Niobate Rapid Adiabatic Couplers Enabling Highly Visible Two‐Photon Interference,” Photonics Research 13, no. 6 (2025): 1579–1590.

[43] H. Heo , M. K. Woo , C.‐H. Park , et al., “On‐Chip Quantum Key Distribution over Field‐Deployed Fiber Using Lithium Niobate Photonic Circuit,” APL Photonics 10, no. 3 (2025): 031301.

[44] C. Wang , M. Zhang , M. Yu , R. Zhu , H. Hu , and M. Loncar , “Monolithic Lithium Niobate Photonic Circuits for Kerr Frequency Comb Generation and Modulation,” Nature Communications 10, no. 1 (2019): 978. 10.1038/s41467-019-08969-6 PMC6395685 30816151

[45] M. Zhang , B. Buscaino , C. Wang , et al., “Broadband Electro‐Optic Frequency Comb Generation in a Lithium Niobate Microring Resonator,” Nature 568, no. 7752 (2019): 373–377. 10.1038/s41586-019-1008-7 30858615

[46] H. Aghaee Rad , T. Ainsworth , R. Alexander , et al., “Scaling and Networking a Modular Photonic Quantum Computer,” Nature 638, no. 8052 (2025): 912–919. 10.1038/s41586-024-08406-9 PMC11864973 39843755

[47] H. S. Stokowski , T. P. McKenna , T. Park , et al., “Integrated Quantum Optical Phase Sensor in Thin‐Film Lithium Niobate,” Nature Communications 14, no. 1 (2023): 3355. 10.1038/s41467-023-38246-6 PMC10250335 37291141

[48] T. Kashiwazaki , T. Yamashima , K. Enbutsu , et al., “Over‐8‐dB Squeezed Light Generation by a Broadband Waveguide Optical Parametric Amplifier Toward Fault‐Tolerant Ultra‐Fast Quantum Computers,” Applied Physics Letters 122, no. 23 (2023): 234003.

[49] A. Kawasaki , H. Brunel , R. Ide , et al., “Real‐Time Observation of Picosecond‐Timescale Optical Quantum Entanglement Towards Ultrafast Quantum Information Processing,” Nature Photonics 19, no. 3 (2025): 271–276.

[50] M. Cerezo , A. Arrasmith , R. Babbush , et al., “Variational Quantum Algorithms,” Nature Reviews Physics 3, no. 9 (2021): 625–644.

[51] A. Peruzzo , J. McClean , P. Shadbolt , et al., “A Variational Eigenvalue Solver on a Photonic Quantum Processor,” Nature Communications 5, no. 1 (2014): 4213. 10.1038/ncomms5213 PMC4124861 25055053

[52] J. Tilly , H. Chen , S. Cao , et al., “The Variational Quantum Eigensolver: A Review of Methods and Best Practices,” Physics Reports 986 (2022): 1–128.

[53] A. Kandala , A. Mezzacapo , K. Temme , et al., “Hardware‐Efficient Variational Quantum Eigensolver for Small Molecules and Quantum Magnets,” Nature 549, no. 7671 (2017): 242–246. 10.1038/nature23879 28905916

[54] T.‐C. Yen , V. Verteletskyi , and A. F. Izmaylov , “Measuring All Compatible Operators in One Series of Single‐Qubit Measurements Using Unitary Transformations,” Journal of Chemical Theory and Computation 16, no. 4 (2020): 2400–2409. 10.1021/acs.jctc.0c00008 32150412

[55] A. F. Izmaylov , T.‐C. Yen , R. A. Lang , and V. Verteletskyi , “Unitary Partitioning Approach to the Measurement Problem in the Variational Quantum Eigensolver Method,” Journal of Chemical Theory and Computation 16, no. 1 (2019): 190–195. 10.1021/acs.jctc.9b00791 31747266

[56] P. Gokhale , O. Angiuli , Y. Ding , et al., “Minimizing State Preparations in Variational Quantum Eigensolver by Partitioning into Commuting Families,” *arXiv* (2019): arXiv:1907.13623.

[57] V. Verteletskyi , T.‐C. Yen , and A. F. Izmaylov , “Measurement Optimization in the Variational Quantum Eigensolver Using a Minimum Clique Cover,” The Journal of Chemical Physics 152, no. 12 (2020): 124114. 10.1063/1.5141458 32241154

[58] I. Hamamura and T. Imamichi , “Efficient Evaluation of Quantum Observables Using Entangled Measurements,” npj Quantum Information 6, no. 1 (2020): 56.

[59] S. Niu , E. Kökcü , S. Johri , et al., “A Practical Framework for Assessing the Performance of Observable Estimation in Quantum Simulation,” *arXiv* (2025): arXiv:2504.09813.

[60] M. Ramôa , P. G. Anastasiou , L. P. Santos , N. J. Mayhall , E. Barnes , and S. E. Economou , “Reducing the Resources Required by ADAPT‐VQE Using Coupled Exchange Operators and Improved Subroutines,” npj Quantum Information 11, no. 1 (2025): 86.

[61] O. Crawford , B. van Straaten , D. Wang , T. Parks , E. Campbell , and S. Brierley , “Efficient Quantum Measurement of Pauli Operators in the Presence of Finite Sampling Error,” Quantum 5 (2021): 385.

[62] T.‐C. Yen , A. Ganeshram , and A. F. Izmaylov , “Deterministic Improvements of Quantum Measurements with Grouping of Compatible Operators, Non‐Local Transformations, and Covariance Estimates,” npj Quantum Information 9, no. 1 (2023): 14. 10.1038/s41534-023-00683-y PMC11041696 38665255

[63] J. M. Turney , A. C. Simmonett , R. M. Parrish , et al., “Psi4: An Open‐Source Ab Initio Electronic Structure Program,” Wiley Interdisciplinary Reviews: Computational Molecular Science 2, no. 4 (2012): 556–565.

[64] Y. Yang , R. J. Chapman , B. Haylock , et al., “Programmable High‐Dimensional Hamiltonian in a Photonic Waveguide Array,” Nature Communications 15, no. 1 (2024): 50. 10.1038/s41467-023-44185-z PMC10761861 38167664

[65] N. Skryabin , I. Kondratyev , I. Dyakonov , O. Borzenkova , S. Kulik , and S. Straupe , “Two‐Qubit Quantum Photonic Processor Manufactured by Femtosecond Laser Writing,” Applied Physics Letters 122, no. 12 (2023): 121102.

[66] O. Borzenkova , G. Struchalin , I. Kondratyev , et al., “Error‐Mitigated Variational Algorithm on a Photonic Processor,” Optics Letters 49, no. 15 (2024): 4453–4456. 10.1364/OL.532017 39090957

[67] N. Maring , A. Fyrillas , M. Pont , et al., “A Versatile Single‐Photon‐Based Quantum Computing Platform,” Nature Photonics 18, no. 6 (2024): 603–609.

[68] K. Powell , X. Li , D. Assumpcao , L. S. Magalhães , N. Sinclair , and M. Lončar , “DC‐Stable Electro‐Optic Modulators Using Thin‐Film Lithium Tantalate,” Optics Express 32, no. 25 (2024): 44115–44122, 10.1364/OE.538870.

[69] J. Holzgrafe , E. Puma , R. Cheng , et al., “Relaxation of the Electro‐Optic Response in Thin‐Film Lithium Niobate Modulators,” Optics Express 32, no. 3 (2024): 3619–3631, 10.1364/OE.507536. 38297579

