# Supplementary material for: Lithium Niobate Electro‐Optic Photonic Processor for Variational Quantum Eigensolver
Source: Adv Sci (Weinh). 2026 Aug 18;13(59):e76876. doi: 10.1002/advs.76876 (PMC13482944; doi:10.1002/advs.76876)
Supplement: Supplementary file 1 [file ADVS-13-e76876-s001.pdf]

Supplementary Information: Lithium Niobate Electro-Optic Photonic Processor for Variational Quantum Eigensolver

Jinil Lee,[1,2] Ui Joon Park,[1,3] Minho Choi,[1,4] Hyeong-Soon Jang,[1] Sunghyun Moon,[1] Hyeon Hwang,[5] Min-Kyo Seo,[5] Dae-Hwan Ahn,[1,6] Sang-Wook Han,[1,6,7] Yong-Su Kim,[1,6] Hyounghan Kwon,[1,6,*] and Hojoong Jung[8,†]

[1]*Center for Quantum Technology, Korea Institute of Science and Technology (KIST), Seoul 02792, South Korea*

[2]*Division of Nano & Information Technology, KIST School, Korea University of Science and Technology, Seoul 02792, South Korea*

[3]*School of Electrical Engineering, Korea University, Seoul 02841, South Korea*

[4]*Department of Artificial Intelligence Semiconductor Engineering, Hanyang University, Seoul, 04763, South Korea*

[5]*Department of Physics, Korea Advanced Institute of Science and Technology (KAIST), Daejeon, 34141, South Korea*

[6]*Division of Quantum Information, KIST School, Korea University of Science and Technology, Seoul 02792, South Korea*

[7]*KU-KIST Graduate School of Converging Science and Technology, Korea University, Seoul 02841, South Korea*

[8]*Department of Physics, Kyung Hee University, Seoul 02447, South Korea*

SUPPLEMENTARY NOTE 1: UNBALANCED MACH–ZEHNDER CHARACTERIZATION OF THE MMI BEAM SPLITTERS

We tested the 2×2 MMI beam splitters using an unbalanced Mach–Zehnder interferometer (MZI). Continuous wave (CW) tunable laser was coupled into and out of the chip using lensed fibers, and the measured transmission spectrum is shown in Supplementary Figure S1. From the spectrum, the transmission at local maximum (1553.34 nm) and minimum (1558.69 nm) was −7.051 dB and −39.780 dB, respectively, which gives an extinction ratio (ER) of,

$$\mathrm{ER} = P_{\max} - P_{\min} = 32.73\ \mathrm{dB},$$

The corresponding fringe visibility is,

$$\mathcal{V} = \frac{P_{\max} - P_{\min}}{P_{\max} + P_{\min}} = \frac{1-r}{1+r}, \qquad r = 10^{-\mathrm{ER}/10} \approx 5.36 \times 10^{-4}, \ \ \mathcal{V} \approx 0.999.$$

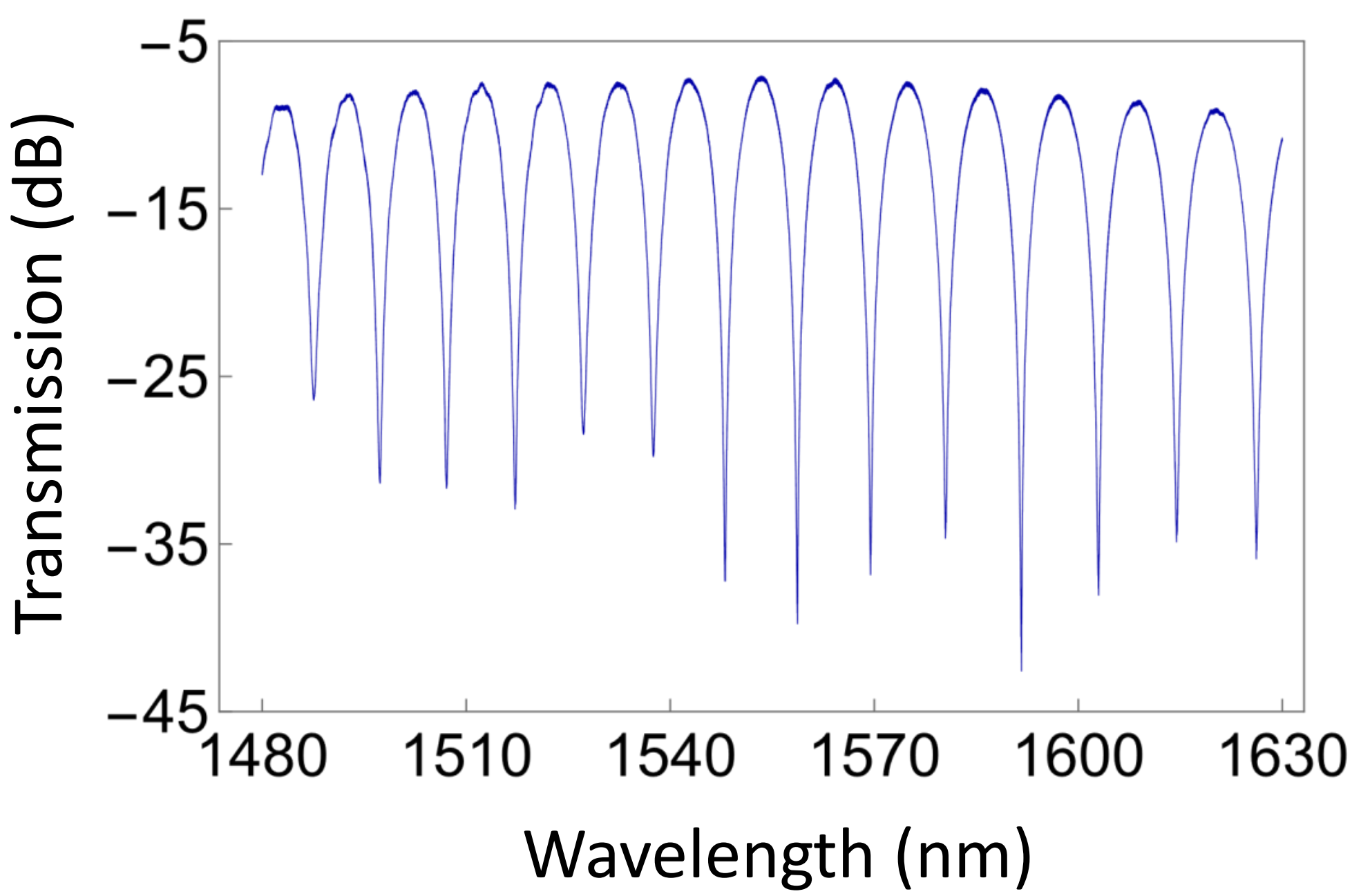

Supplementary Figure S1 | Unbalanced Mach–Zehnder spectrum used to characterize the MMI beam splitters. Measured transmission versus wavelength of the test interferometer consists of two identical MMIs.

* hyounghankwon@kist.re.kr; Co-corresponding author

† hojoong.jung@khu.ac.kr; Co-corresponding author

This high ER indicates that the MMI couplers operate very close to the 50/50 splitting condition around the telecom band. Currently, the ER is mainly limited by the input polarization alignment.

SUPPLEMENTARY NOTE 2: TWELVE-CHANNEL ELECTRO-OPTIC MODULATION

We characterized all twelve electro-optic (EO) phase shifters on the ququart processor by applying a linear voltage sweep to each element at a wavelength of 1560 nm. For each channel, the bias was swept from −28 V to +28 V and the transmitted power at one output port was recorded on a photodetector. The resulting Mach–Zehnder interference fringes were used to extract the half-wave voltage V_π. The set of V_π values for $\{\theta_1, \ldots, \theta_6, \phi_1, \ldots, \phi_6\}$ is summarized in Supplementary Figure S2 in log scale.

During algorithm runs, the EO elements were driven by a National Instruments data-acquisition unit with synchronized analog outputs and high-voltage amplifiers supplying ±28 V to the electrodes.

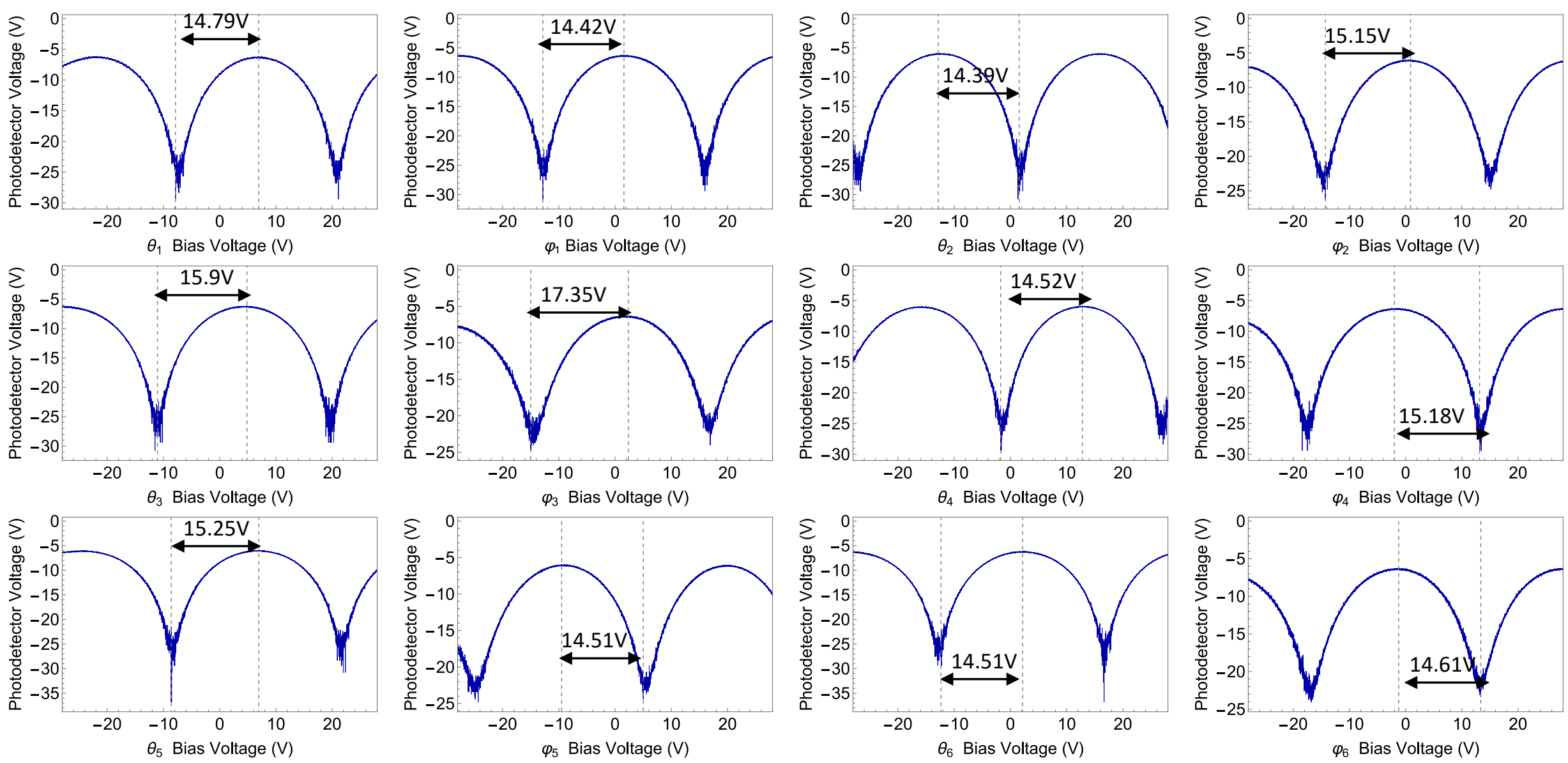

Supplementary Figure S2 | Linear voltage sweep characterization of the twelve EO channels in log scale. Each panel shows photodetector signal versus applied bias for one phase shifter. The half-wave voltage V_π is obtained from the consecutive extrema and is annotated in each subplot.

SUPPLEMENTARY NOTE 3: DC DRIFT AND FIBER–TO–CHIP COUPLING STABILITY

We tested the DC drift of on-chip electro-optic (EO) phase shifters under constant bias. All EO phase shifters were at fixed DC voltages and the measured optical powers were maintained at constant values, indicating that there was no DC drift from the EO phase shifters. The input and output fibers were aligned with NanoMax 300 closed-loop piezo stages that provide stable fiber–to–chip coupling during the 50 min measurement time.

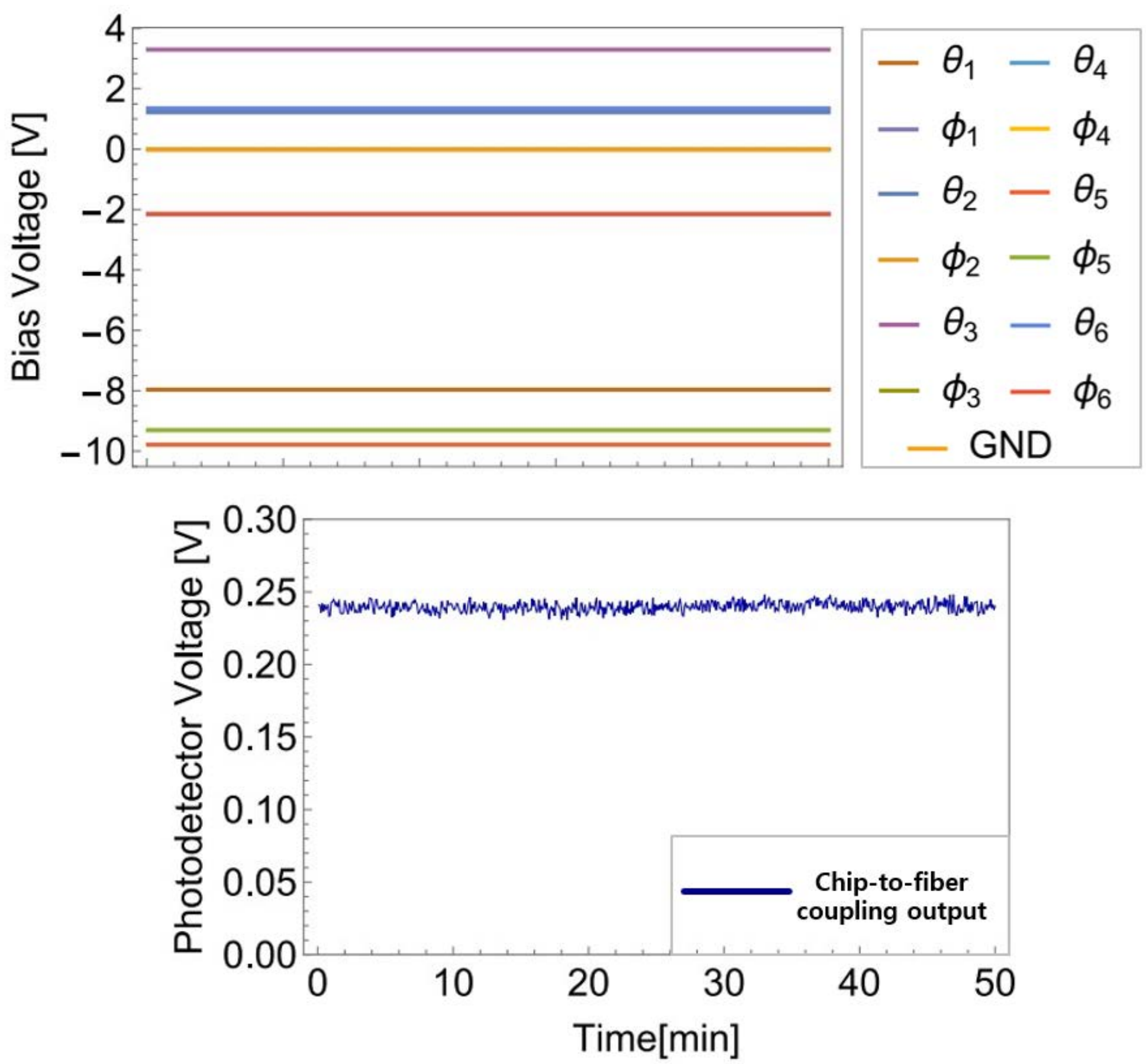

Supplementary Figure S3 | DC-drift measurement under fixed DC biases. Fiber–to–chip coupling was maintained by a NanoMax 300 closed-loop piezo stage.

SUPPLEMENTARY NOTE 4: QUANTUM STATE TOMOGRAPHY OF ON-CHIP QUQUART STATES

We validated on-chip state preparation and measurement by performing full quantum state tomography (QST) of ququart states using the LNOI processor. States were prepared by electrically tuning the Mach–Zehnder interferometers and EO phase shifters. Projective measurements for tomography were implemented on the same chip by reconfiguring the circuit to the required 36 measurement states. For each setting, we integrated single-photon counts under the charge-balanced drive condition shown in Figure 2(d). Density matrices were reconstructed with a maximum-likelihood estimator. For a target pure state $|\psi_t\rangle$ we report the fidelity $F = \langle\psi_t|\,\hat{\rho}\,|\psi_t\rangle$ and the purity $P = \mathrm{Tr}(\hat{\rho}^2)$. Across all prepared states, we observed an average fidelity of 98.2% and an average purity of 98.4%, as shown in Supplementary Figure S4.

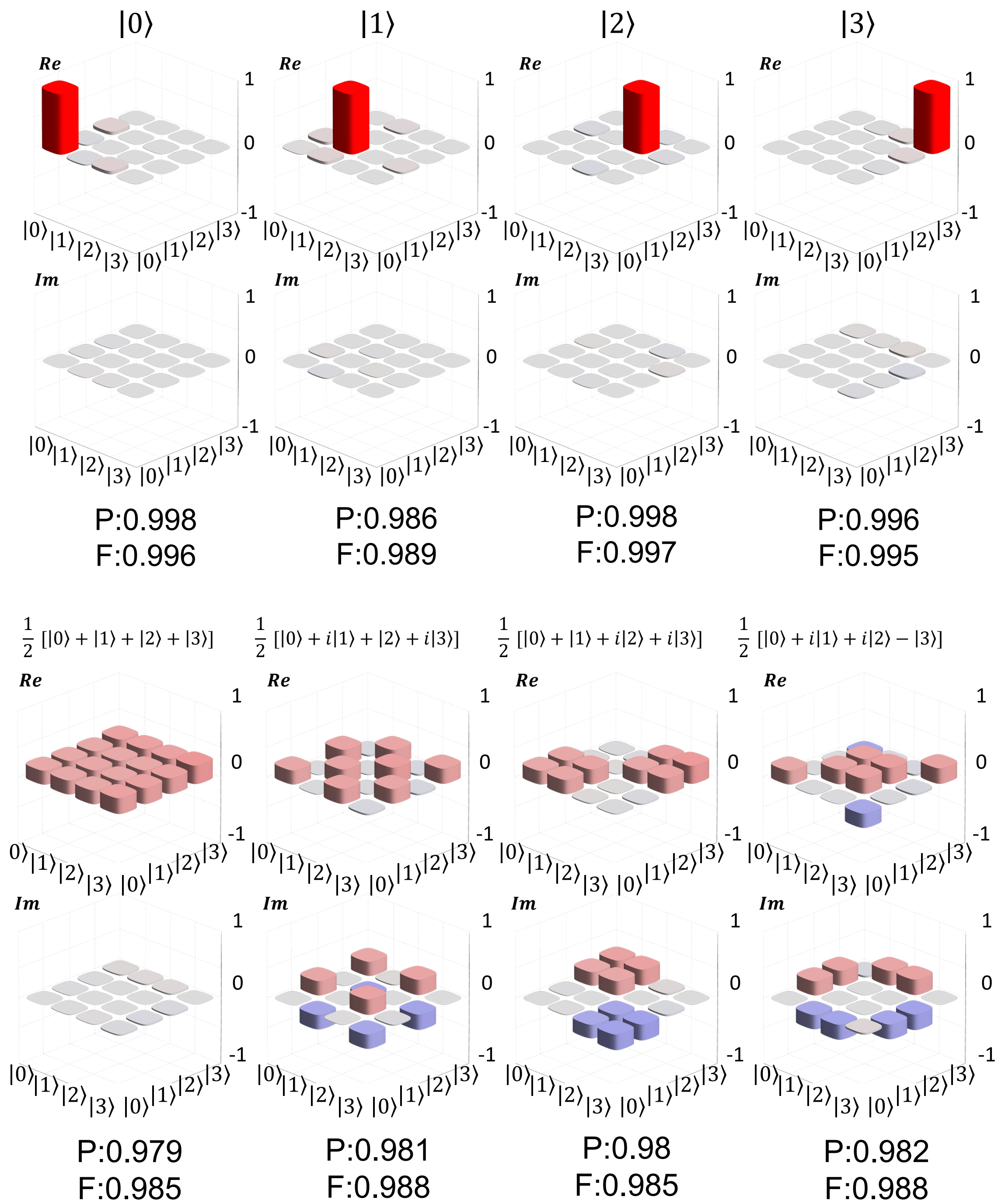

|0⟩
|1⟩
|2⟩
|3⟩
Re
Im
P:0.998
F:0.996
P:0.986
F:0.989
P:0.998
F:0.997
P:0.996
F:0.995
$\frac{1}{2}[|0\rangle + |1\rangle + |2\rangle + |3\rangle]$
$\frac{1}{2}[|0\rangle + i|1\rangle + |2\rangle + i|3\rangle]$
$\frac{1}{2}[|0\rangle + |1\rangle + i|2\rangle + i|3\rangle]$
$\frac{1}{2}[|0\rangle + i|1\rangle + i|2\rangle - |3\rangle]$
P:0.979
F:0.985
P:0.981
F:0.988
P:0.98
F:0.985
P:0.982
F:0.988

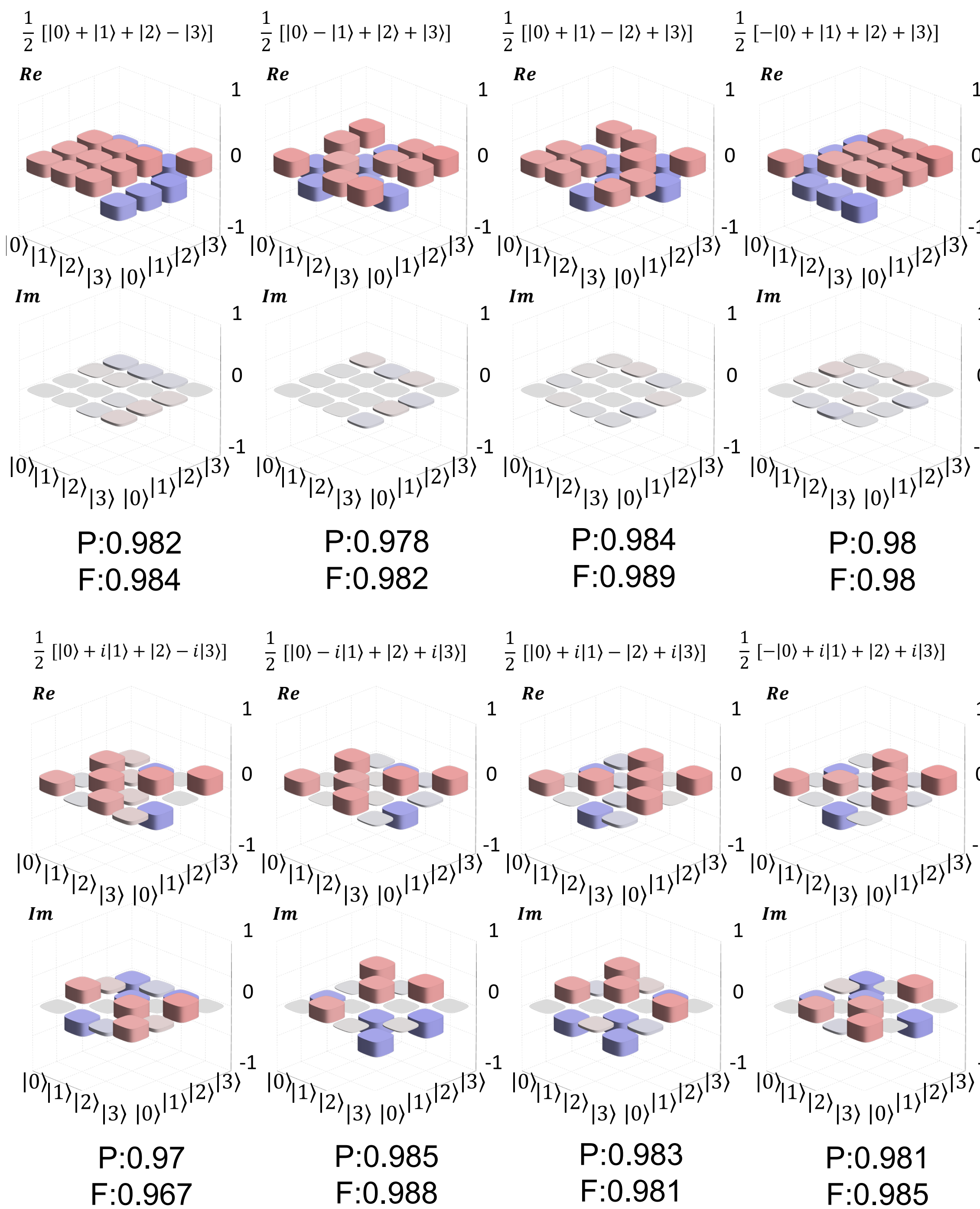

$\frac{1}{2}[|0\rangle + |1\rangle + |2\rangle - |3\rangle]$
$\frac{1}{2}[|0\rangle - |1\rangle + |2\rangle + |3\rangle]$
$\frac{1}{2}[|0\rangle + |1\rangle - |2\rangle + |3\rangle]$
$\frac{1}{2}[-|0\rangle + |1\rangle + |2\rangle + |3\rangle]$
Re
Im
P:0.982
F:0.984
P:0.978
F:0.982
P:0.984
F:0.989
P:0.98
F:0.98
$\frac{1}{2}[|0\rangle + i|1\rangle + |2\rangle - i|3\rangle]$
$\frac{1}{2}[|0\rangle - i|1\rangle + |2\rangle + i|3\rangle]$
$\frac{1}{2}[|0\rangle + i|1\rangle - |2\rangle + i|3\rangle]$
$\frac{1}{2}[-|0\rangle + i|1\rangle + |2\rangle + i|3\rangle]$
Re
Im
P:0.97
F:0.967
P:0.985
F:0.988
P:0.983
F:0.981
P:0.981
F:0.985

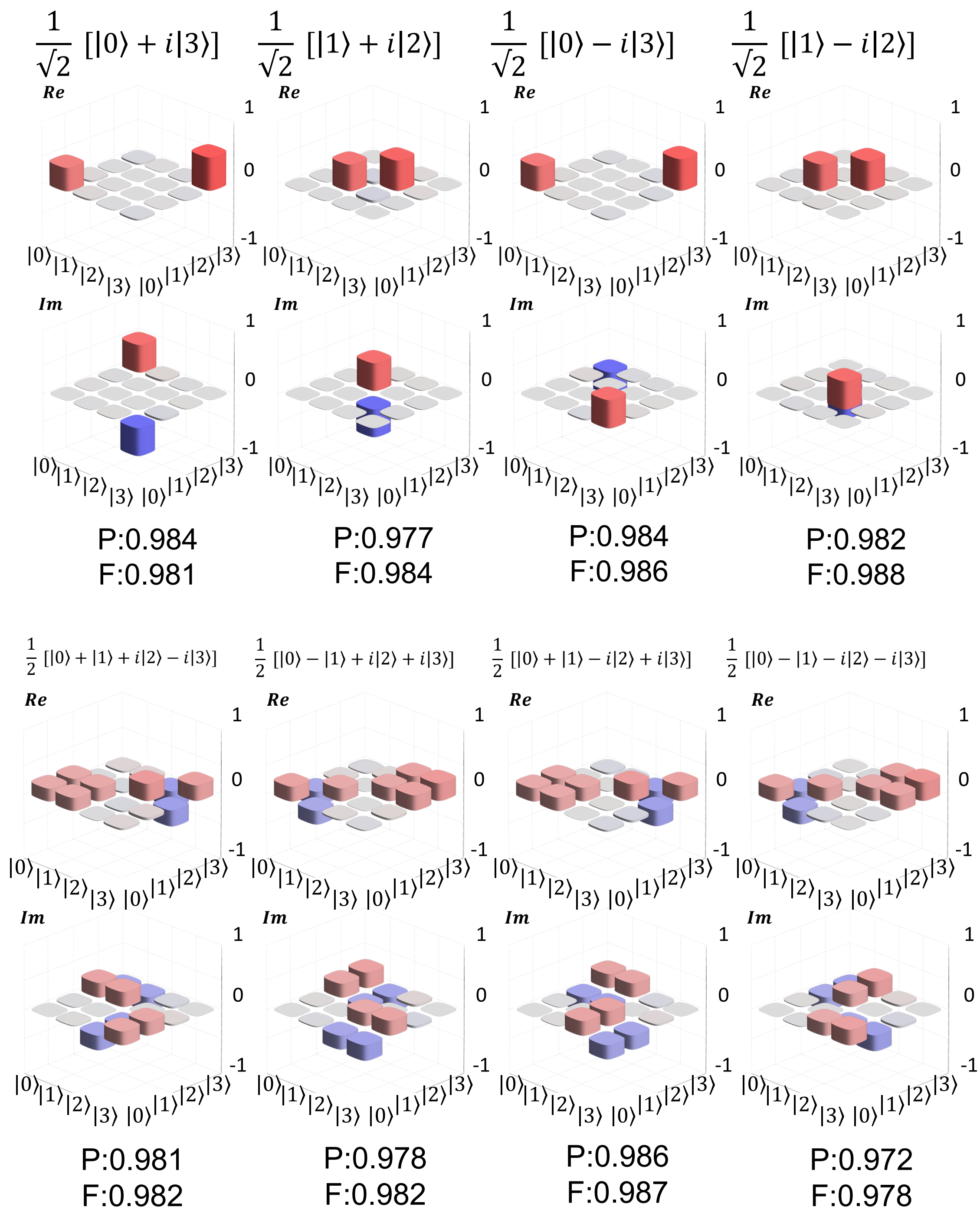

$\frac{1}{\sqrt{2}}[|0\rangle + i|3\rangle]$
$\frac{1}{\sqrt{2}}[|1\rangle + i|2\rangle]$
$\frac{1}{\sqrt{2}}[|0\rangle - i|3\rangle]$
$\frac{1}{\sqrt{2}}[|1\rangle - i|2\rangle]$
Re
Im
P:0.984
F:0.981
P:0.977
F:0.984
P:0.984
F:0.986
P:0.982
F:0.988
$\frac{1}{2}[|0\rangle + |1\rangle + i|2\rangle - i|3\rangle]$
$\frac{1}{2}[|0\rangle - |1\rangle + i|2\rangle + i|3\rangle]$
$\frac{1}{2}[|0\rangle + |1\rangle - i|2\rangle + i|3\rangle]$
$\frac{1}{2}[|0\rangle - |1\rangle - i|2\rangle - i|3\rangle]$
P:0.981
F:0.982
P:0.978
F:0.982
P:0.986
F:0.987
P:0.972
F:0.978

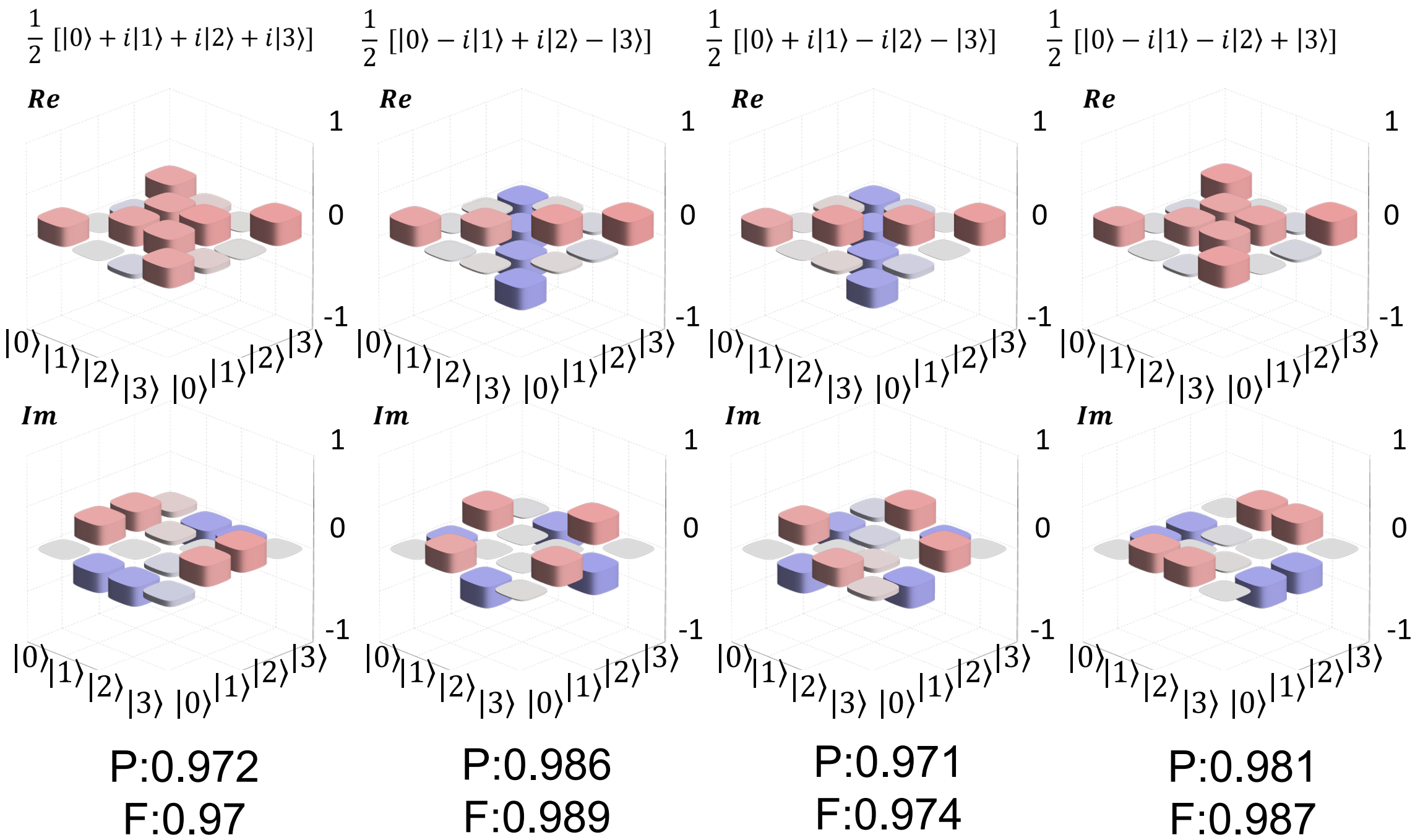

Supplementary Figure S4 | Quantum state tomography (QST) of on-chip ququart states. Reconstructed density matrices (real and imaginary parts) for representative states, with fidelity F and purity P listed in each panel.

SUPPLEMENTARY NOTE 5: SENSITIVITY OF THE CHEMICAL-ACCURACY ASSESSMENT TO THE NUMBER OF LOWEST-ENERGY POINTS AVERAGED

To evaluate how sensitive the chemical-accuracy assessment is to the number of lowest-energy points averaged, we repeated the analysis using $K = 5, 10$, and 15, and calculated the absolute deviation $|E_{\text{lowest } K} - E_{\text{theory}}|$ at each interatomic distance.

Supplementary Figure S5 shows the resulting deviation in units of mHa. The $K = 5$ curve remains within the chemical-accuracy threshold of 1.6 mHa for all measured interatomic distances, with a maximum deviation of 1.397 mHa. Increasing K includes additional higher-energy points from the convergence region and can move some distances outside the threshold. This analysis shows how the chemical-accuracy estimate depends on the averaging window.

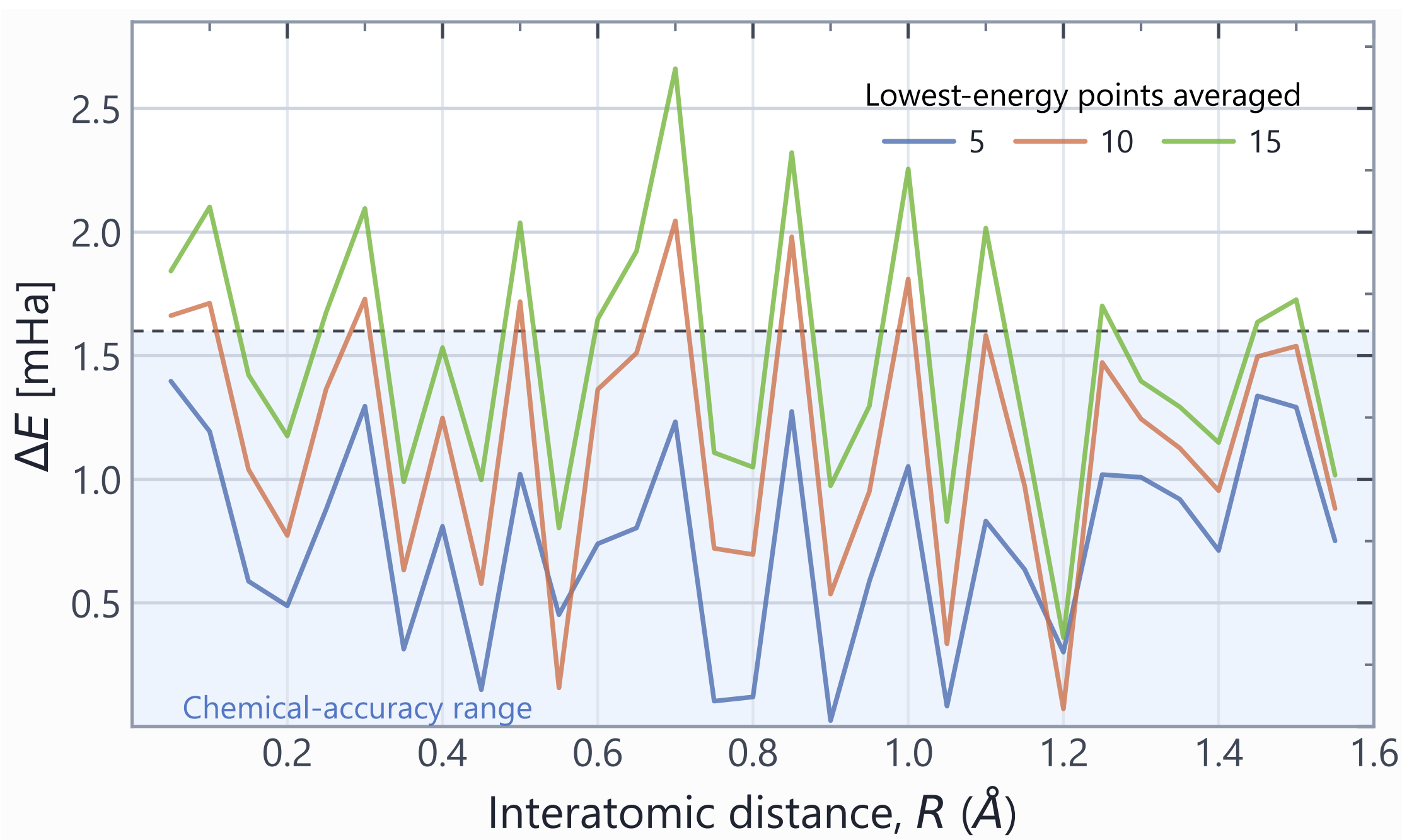

Supplementary Figure S5 | Sensitivity of the chemical-accuracy assessment to the number of lowest-energy points averaged. The shaded region indicates the 1.6 mHa chemical-accuracy threshold. The $K = 5$ case remains within chemical accuracy for all measured interatomic distances.

SUPPLEMENTARY NOTE 6: CHARACTERIZATION OF THE INTEGRATED PPLNOI QUQUART PROCESSOR

All SHG/SPDC characterization, PPLNOI-source QST, and source-integrated VQE measurements were performed on the same source-integrated PPLNOI processor shown in main-text Fig. 5(a), not on a separate test structure. We first characterized the periodically poled LNOI (PPLNOI) source by sweeping the pump wavelength around the quasi-phase-matched region using a near-visible tunable laser (TOPTICA CTL 780) and recording the single counts and coincidence counts from the integrated PPLNOI source. The PPLNOI source was designed with a 3.2 μm poling period. Supplementary Figure S7 shows a second-harmonic generation (SHG) microscopy image of the periodically poled source section on the same source-integrated PPLNOI processor used for the SHG/SPDC, QST, and VQE measurements. The source-waveguide width was swept from 2.30 μm to 2.40 μm in the design, and 2.36 μm was used as the target width for the measured source waveguide. We first performed the sweep using only the long-pass filter, for which the coincidence counts exhibited a pronounced peak at a pump wavelength of 787.711 nm, as shown in Supplementary Figure S6(a). We then angle-tuned a 1580 nm, 12 nm band-pass filter so that its passband overlapped the degenerate SPDC wavelength. With the angle-tuned band-pass filter, the filtered coincidence response was observed around the operating pump wavelength, as shown in Supplementary Figure S6(b). The QST and VQE measurements reported in the main text were performed with a pump wavelength of 788.25 nm, and 350 μW of off-chip pump power was coupled into the PPLNOI waveguide using a lensed fiber. The device temperature was set to 20°C, slightly below room temperature, to help mitigate DC drift during the measurements. This pump wavelength corresponds to a nominal degenerate SPDC wavelength of 1576.5 nm. For the PPLNOI-source QST and VQE measurements, each projection used a timing sequence in which the EO electrodes were held at 0 V for 0.8 s, the target voltage was applied, photon counts were measured for 0.1 s after a 0.3 s synchronization delay, and an opposite-polarity bias was applied for 0.4 s before the next setting. Estimated accidental coincidences were subtracted before reconstructing projection probabilities.

We next characterized the voltage response of the PPLNOI ququart chip under the coincidence-count measurement condition. Each electro-optic channel was ramped from −30 V to +30 V in 0.1 V steps with a 0.1 s integration time per point, while monitoring the coincidence counts (CC) between D1 and D2. In an LN Mach–Zehnder circuit, the applied voltage induces a Pockels phase

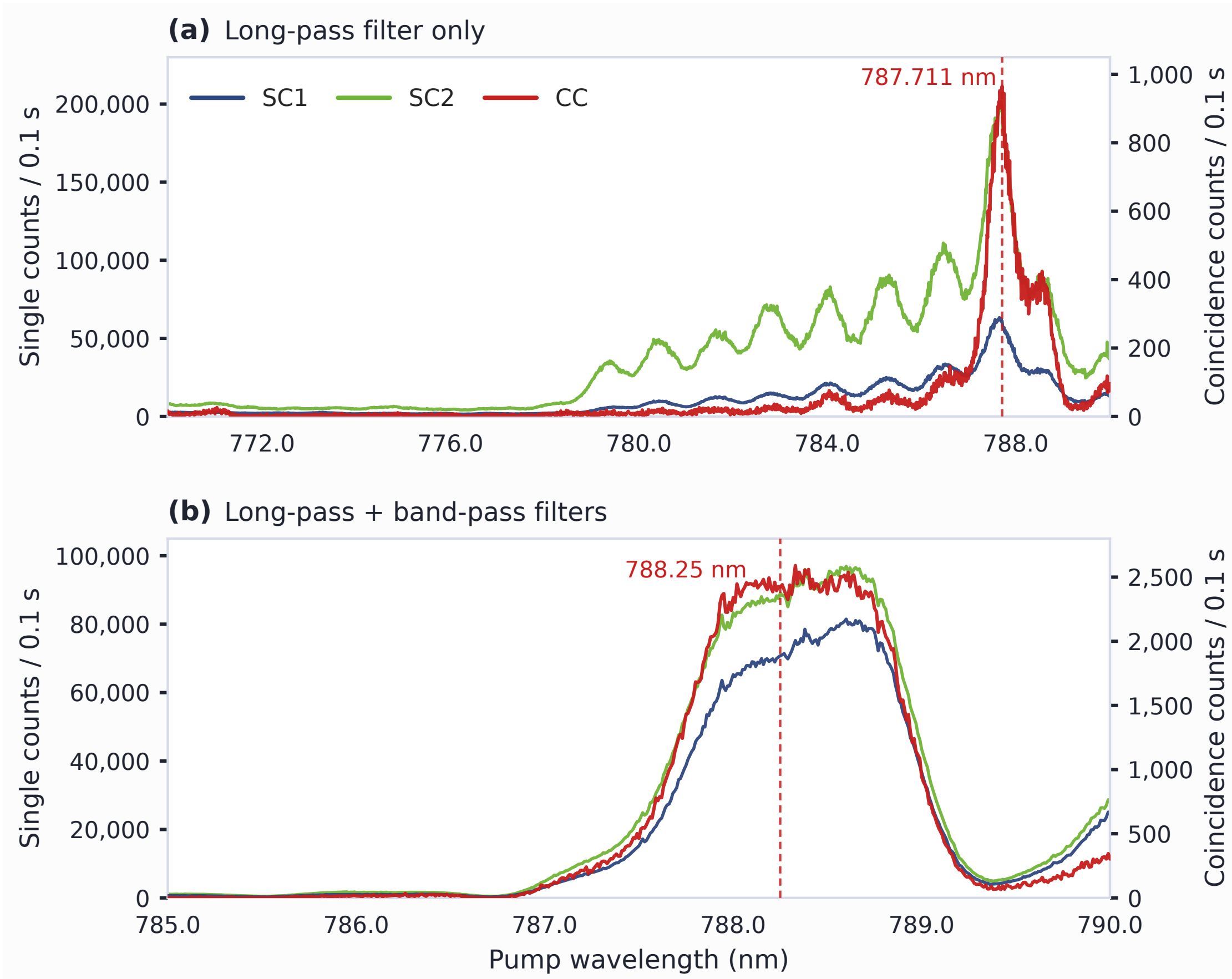

Supplementary Figure S6 | SPDC count sweeps of the integrated PPLNOI photon-pair source. The pump wavelength was swept using a near-visible tunable laser (TOPTICA CTL 780) while recording the single counts at two output channels (SC1 and SC2, left axis) and the coincidence counts (CC, right axis). **(a)** Sweep measured using only the long-pass filter. The dashed line marks the coincidence-count peak at 787.711 nm, in sub-0.1 nm agreement with the expected degenerate SPDC pump wavelength extrapolated from the SHG peak. **(b)** Sweep measured with the long-pass filter and an angle-tuned 1580 nm, 12 nm band-pass filter. The dashed line marks the pump wavelength used for the PPLNOI-source QST and VQE measurements, 788.25 nm, corresponding to a nominal degenerate SPDC wavelength of 1576.5 nm.

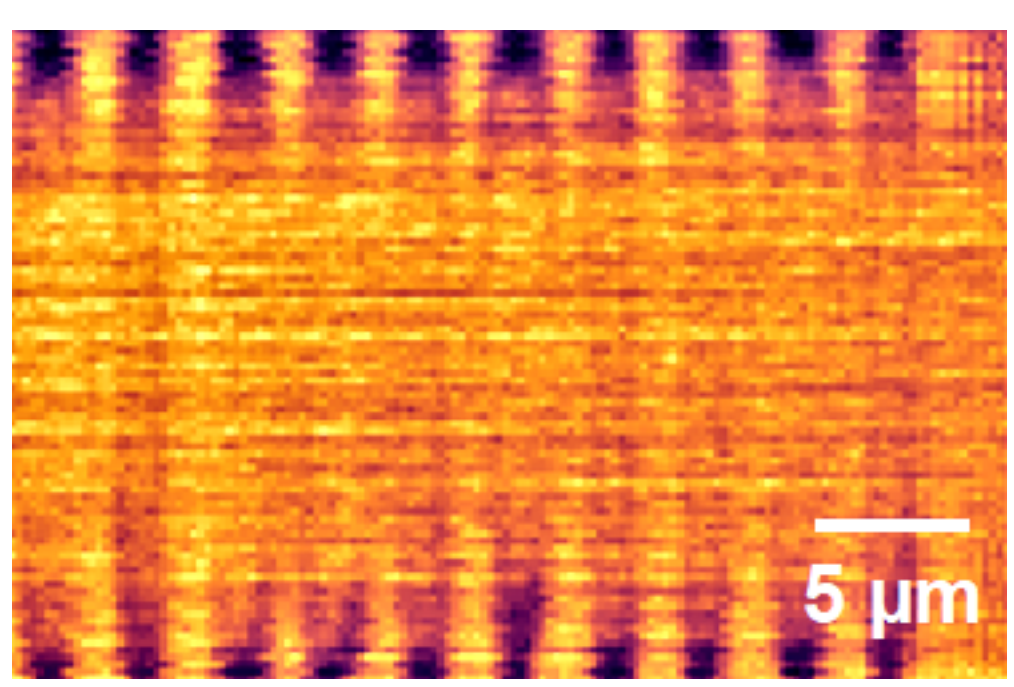

Supplementary Figure S7 | Second-harmonic generation (SHG) microscopy image of the periodically poled PPLNOI source section.

shift, producing a voltage-dependent interference fringe at the output. The observed coincidence-count modulation therefore confirms voltage-controlled EO phase tuning of the integrated PPLNOI circuit under the same integrated-source measurement conditions used for the PPLNOI QST and VQE experiments. The slow DC drift during the sequential voltage ramp is consistent with the deviations from an ideal static fringe.

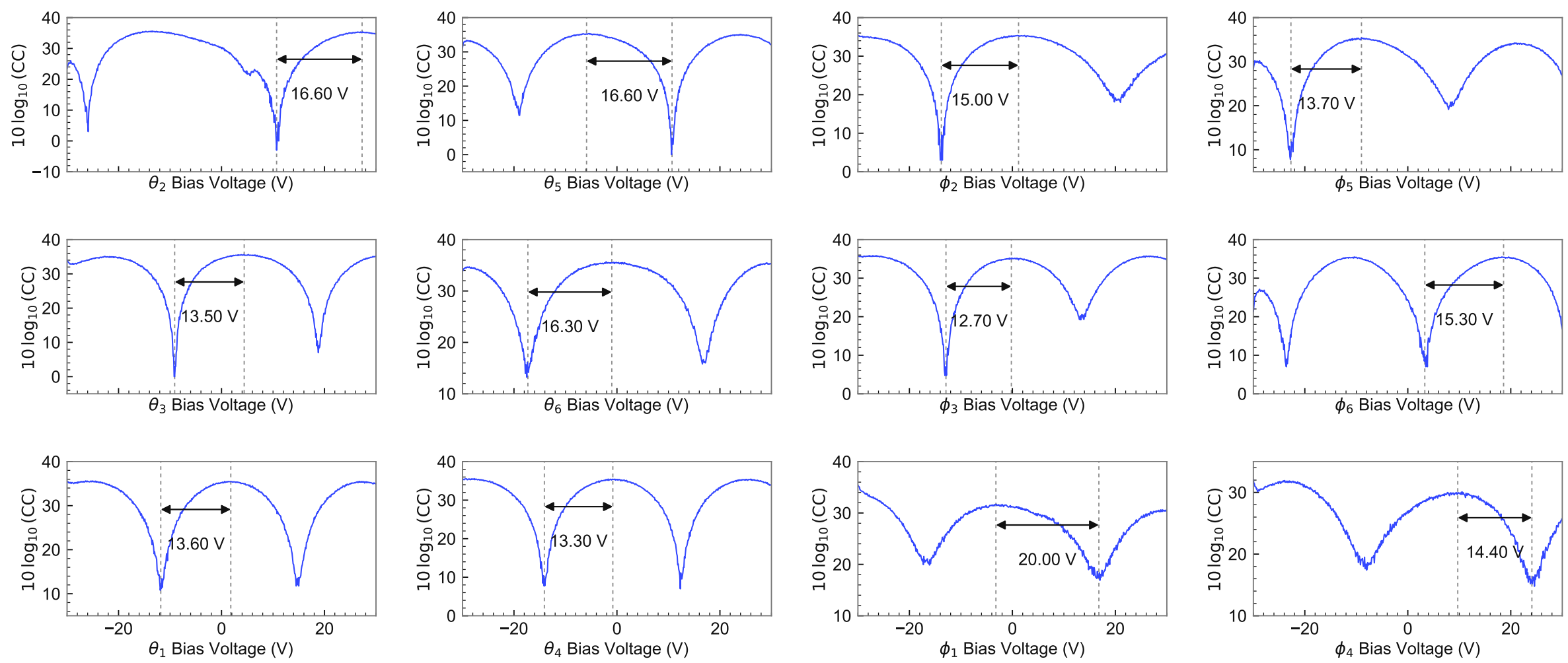

Supplementary Figure S8 | Twelve-channel voltage sweep characterization of the PPLNOI ququart chip using coincidence counts. Each panel shows $10\log_{10}(\mathrm{CC})$ versus applied bias for one θ or ϕ electro-optic channel, acquired while ramping the bias in 0.1 V steps with a 0.1 s integration time per point. The arrows indicate the voltage separation between consecutive extrema used to estimate V_π.

We also monitored the transient coincidence-count response after applying a bias voltage to the same PPLNOI device from the zero-voltage condition. The coincidence-count response shows an initial increase followed by relaxation toward a steady level, as shown in Supplementary Figure S9.

Finally, as in Supplementary Note 4, we performed full quantum state tomography (QST) of ququart states using the PPLNOI device with on-chip SPDC. The states were prepared by electrically tuning the Mach–Zehnder interferometers and electro–optic phase shifters, and tomography used the same set of 36 projective measurements implemented by reconfiguring the circuit. Density matrices were reconstructed with a maximum-likelihood estimator. Representative results are shown in Supplementary Figure S10, and the corresponding fidelities F and purities P are listed in each panel. Across the 40 prepared states, the mean purity and mean fidelity were $P = 0.9829$ and $F = 0.9859$, respectively. These data confirm that the PPLNOI device supports reliable state preparation and measurement with the integrated PPLNOI source.

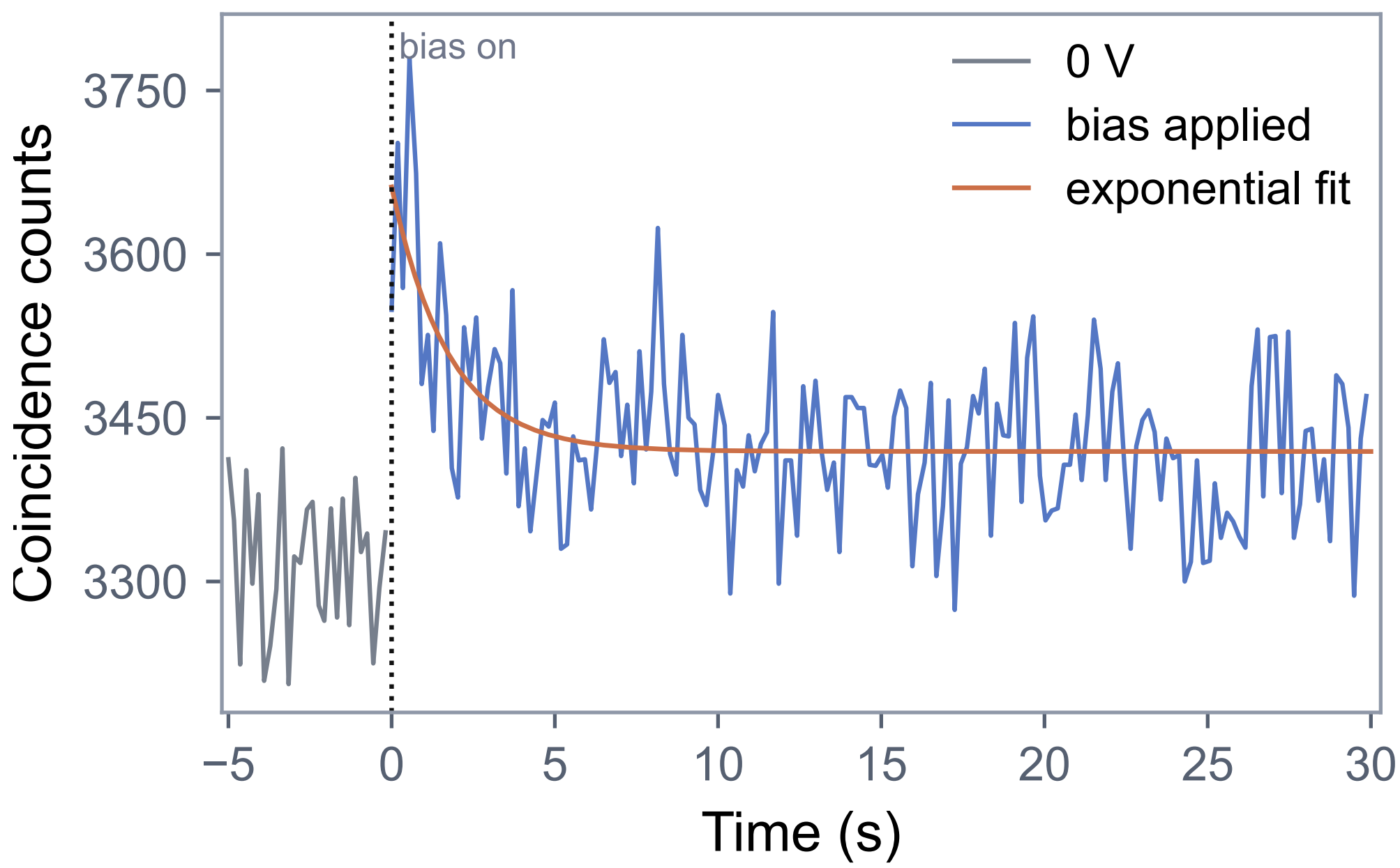

Supplementary Figure S9 | Coincidence-count response after applying a bias voltage to the PPLNOI ququart chip from 0 V. The vertical dotted line marks the bias application time, and the orange curve shows an exponential fit used as a visual guide.

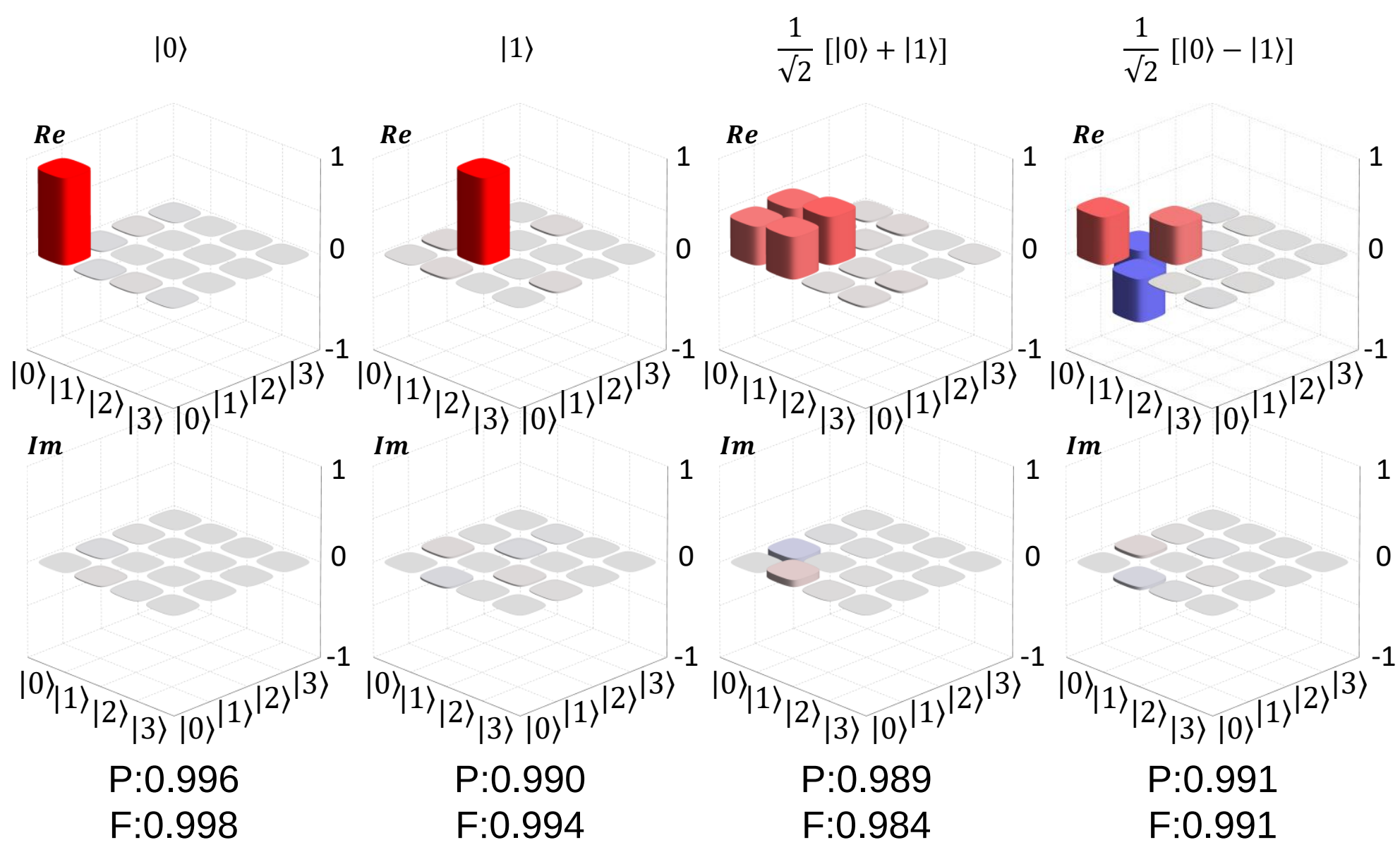
|0⟩
|1⟩
$\frac{1}{\sqrt{2}}[|0\rangle + |1\rangle]$
$\frac{1}{\sqrt{2}}[|0\rangle - |1\rangle]$
Re
Im
P:0.996
F:0.998
P:0.990
F:0.994
P:0.989
F:0.984
P:0.991
F:0.991

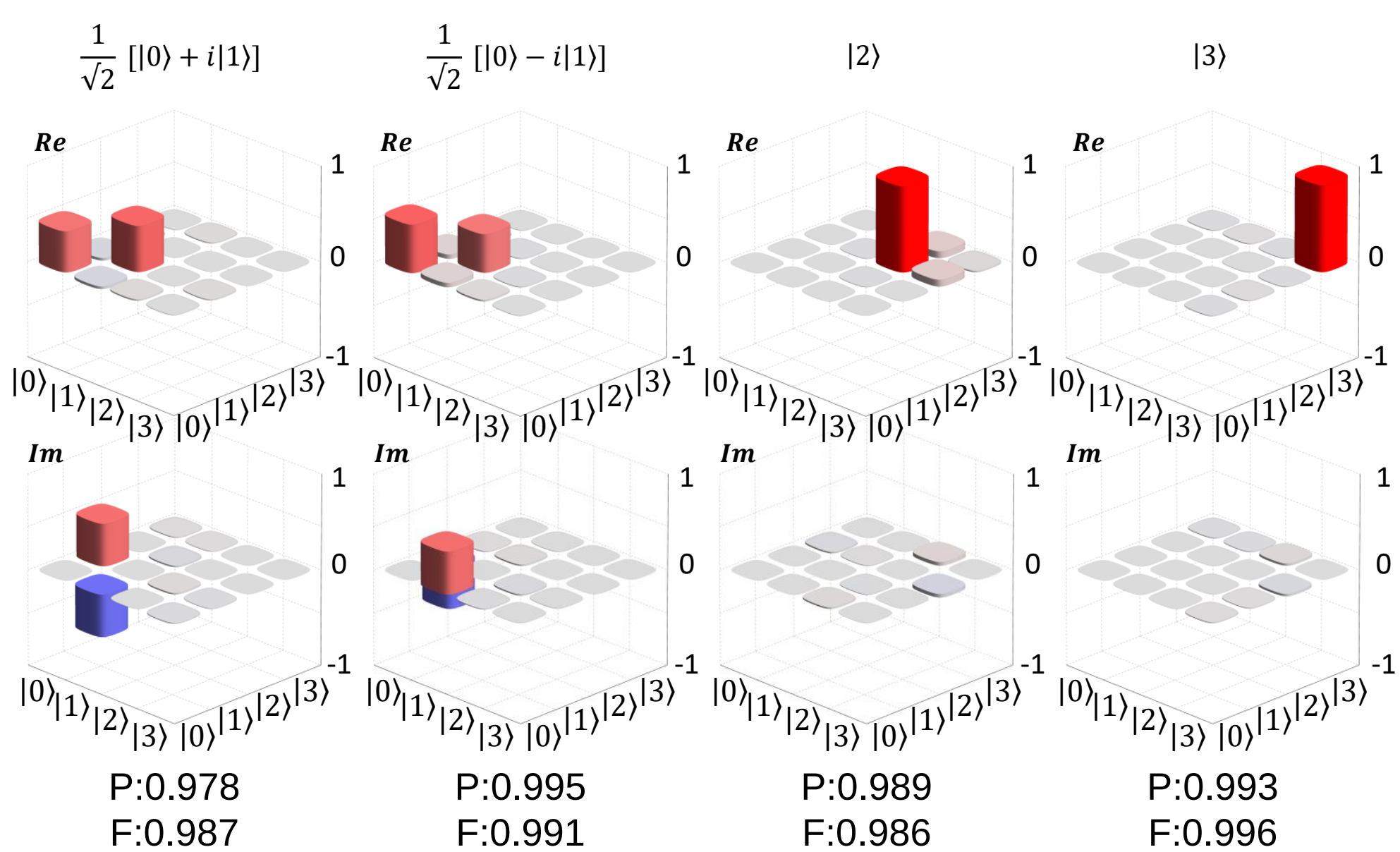
$\frac{1}{\sqrt{2}}[|0\rangle + i|1\rangle]$
$\frac{1}{\sqrt{2}}[|0\rangle - i|1\rangle]$
|2⟩
|3⟩
Re
Im
P:0.978
F:0.987
P:0.995
F:0.991
P:0.989
F:0.986
P:0.993
F:0.996

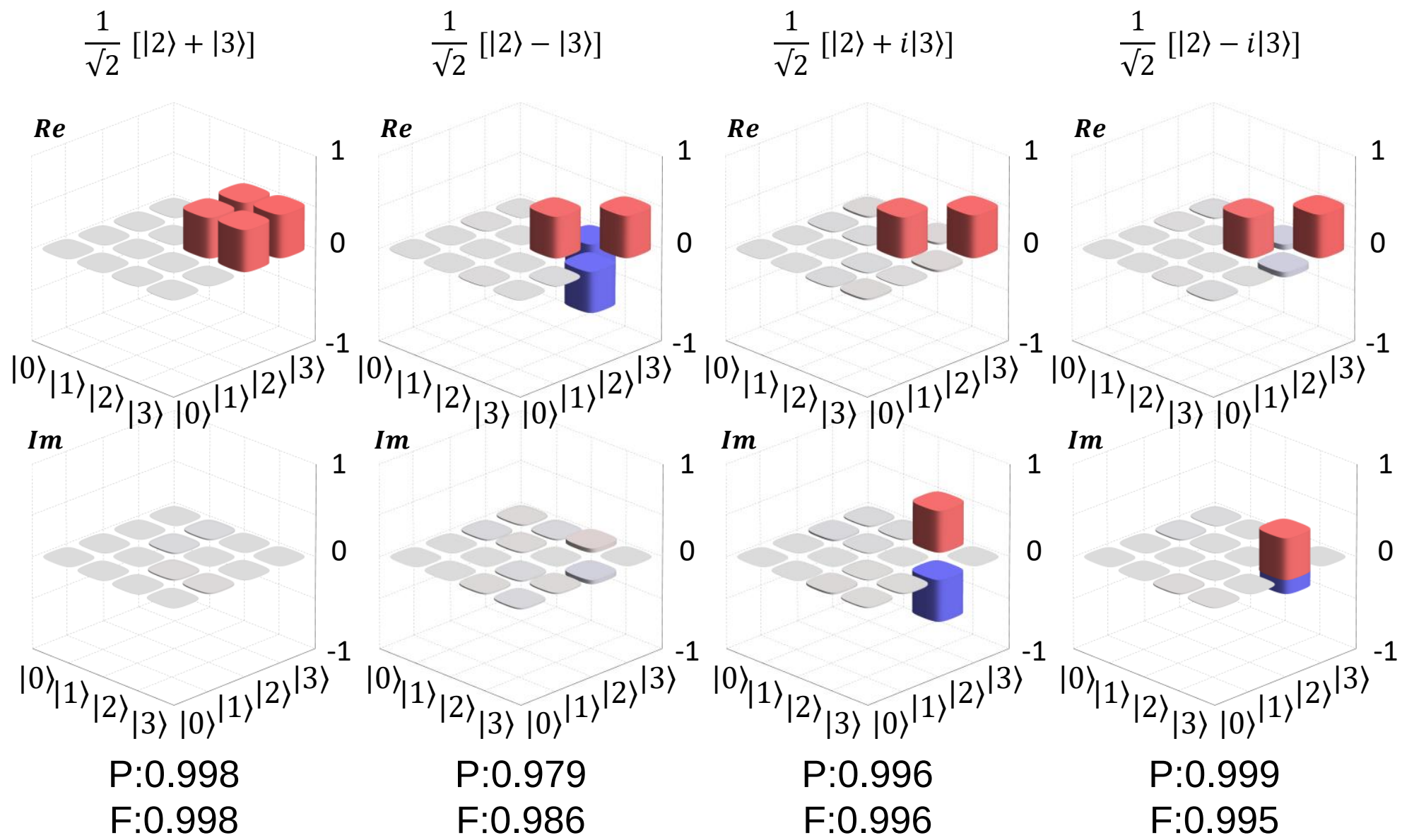
$\frac{1}{\sqrt{2}}[|2\rangle + |3\rangle]$
$\frac{1}{\sqrt{2}}[|2\rangle - |3\rangle]$
$\frac{1}{\sqrt{2}}[|2\rangle + i|3\rangle]$
$\frac{1}{\sqrt{2}}[|2\rangle - i|3\rangle]$
Re
Im
P:0.998
F:0.998
P:0.979
F:0.986
P:0.996
F:0.996
P:0.999
F:0.995

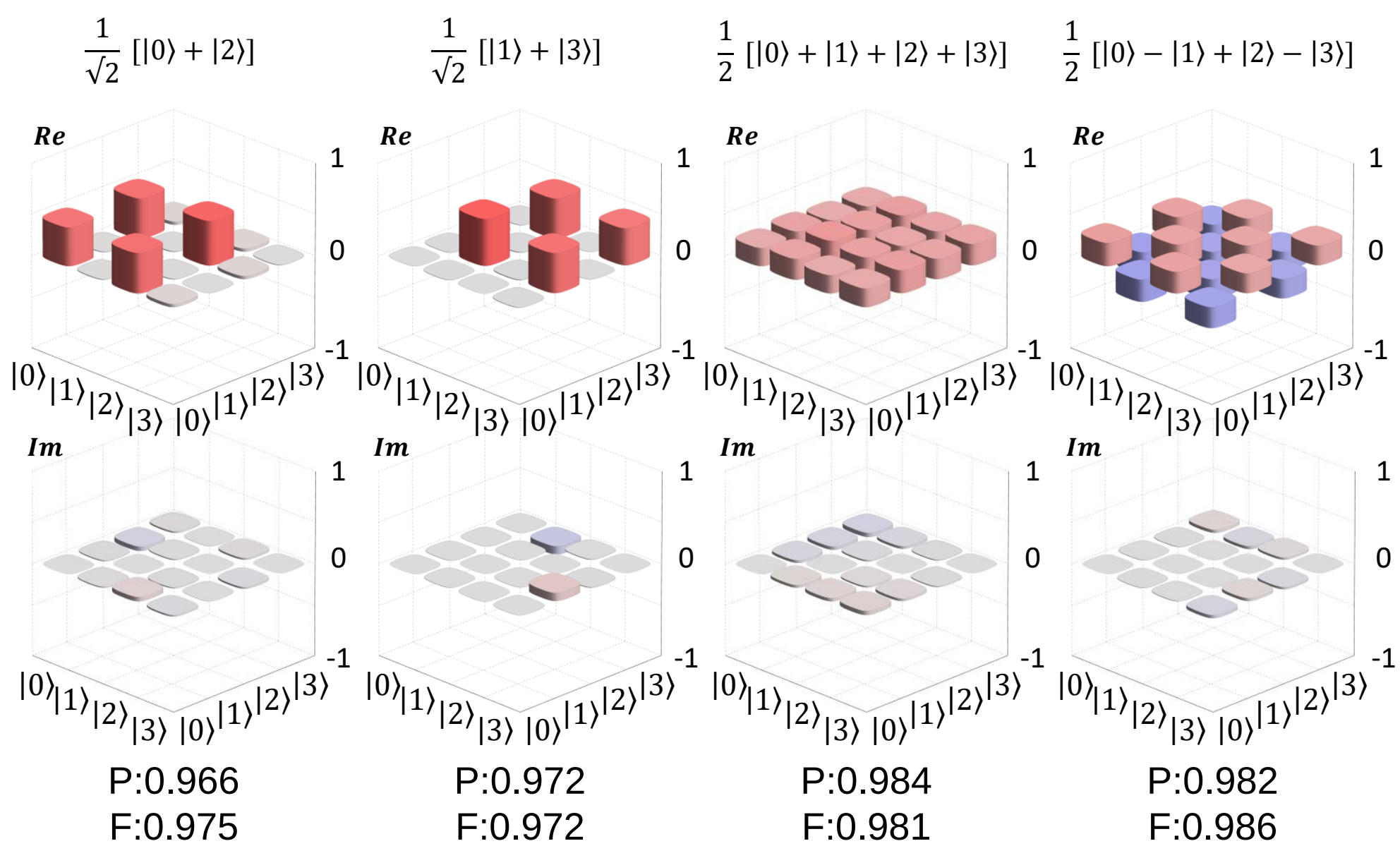
$\frac{1}{\sqrt{2}}[|0\rangle + |2\rangle]$
$\frac{1}{\sqrt{2}}[|1\rangle + |3\rangle]$
$\frac{1}{2}[|0\rangle + |1\rangle + |2\rangle + |3\rangle]$
$\frac{1}{2}[|0\rangle - |1\rangle + |2\rangle - |3\rangle]$
Re
Im
P:0.966
F:0.975
P:0.972
F:0.972
P:0.984
F:0.981
P:0.982
F:0.986

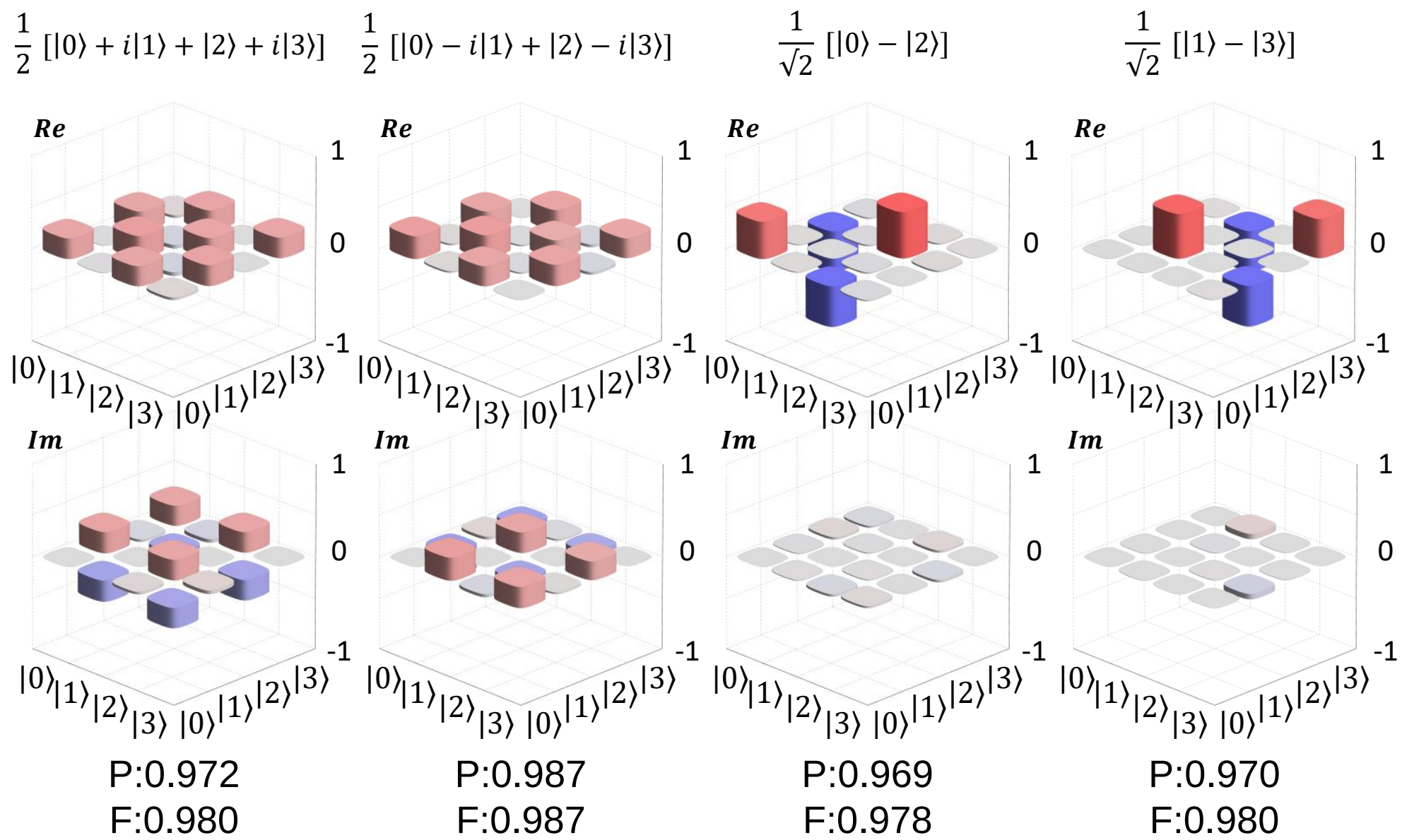
$\frac{1}{2}[|0\rangle + i|1\rangle + |2\rangle + i|3\rangle]$
$\frac{1}{2}[|0\rangle - i|1\rangle + |2\rangle - i|3\rangle]$
$\frac{1}{\sqrt{2}}[|0\rangle - |2\rangle]$
$\frac{1}{\sqrt{2}}[|1\rangle - |3\rangle]$
Re
Im
P:0.972
F:0.980
P:0.987
F:0.987
P:0.969
F:0.978
P:0.970
F:0.980

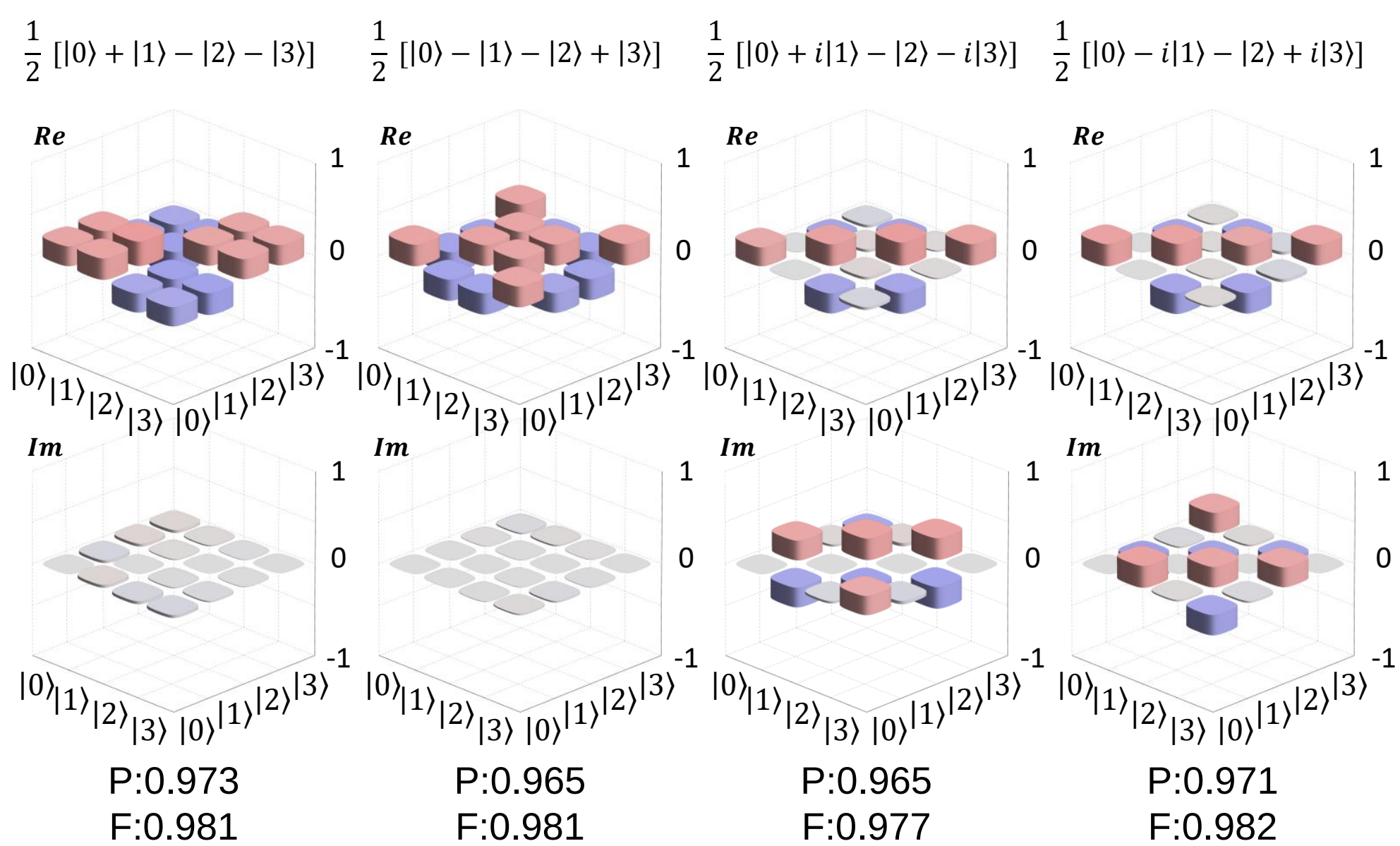
$\frac{1}{2}[|0\rangle + |1\rangle - |2\rangle - |3\rangle]$
$\frac{1}{2}[|0\rangle - |1\rangle - |2\rangle + |3\rangle]$
$\frac{1}{2}[|0\rangle + i|1\rangle - |2\rangle - i|3\rangle]$
$\frac{1}{2}[|0\rangle - i|1\rangle - |2\rangle + i|3\rangle]$
Re
Im
P:0.973
F:0.981
P:0.965
F:0.981
P:0.965
F:0.977
P:0.971
F:0.982

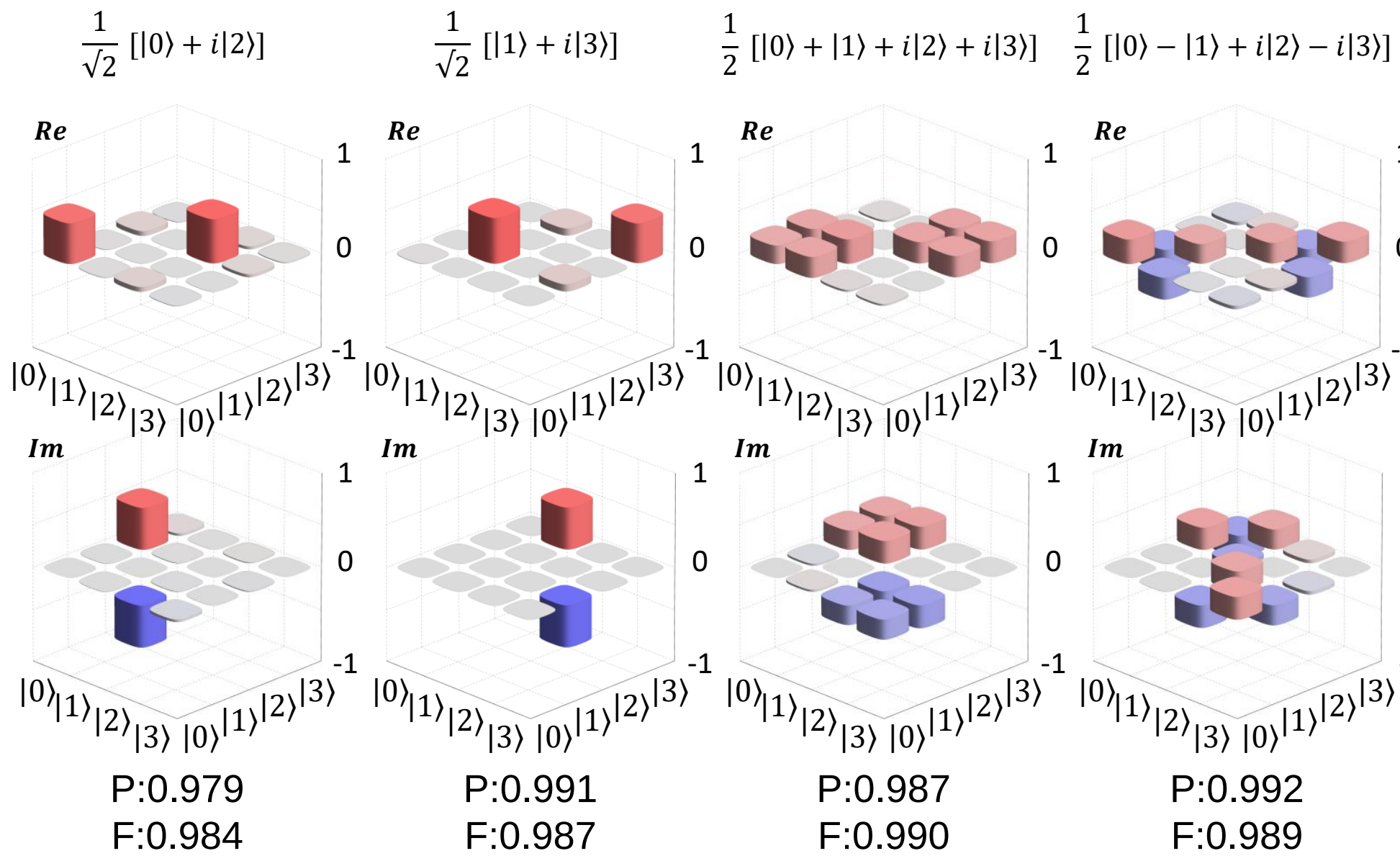
$\frac{1}{\sqrt{2}}[|0\rangle + i|2\rangle]$
$\frac{1}{\sqrt{2}}[|1\rangle + i|3\rangle]$
$\frac{1}{2}[|0\rangle + |1\rangle + i|2\rangle + i|3\rangle]$
$\frac{1}{2}[|0\rangle - |1\rangle + i|2\rangle - i|3\rangle]$
Re
Im
P:0.979
F:0.984
P:0.991
F:0.987
P:0.987
F:0.990
P:0.992
F:0.989

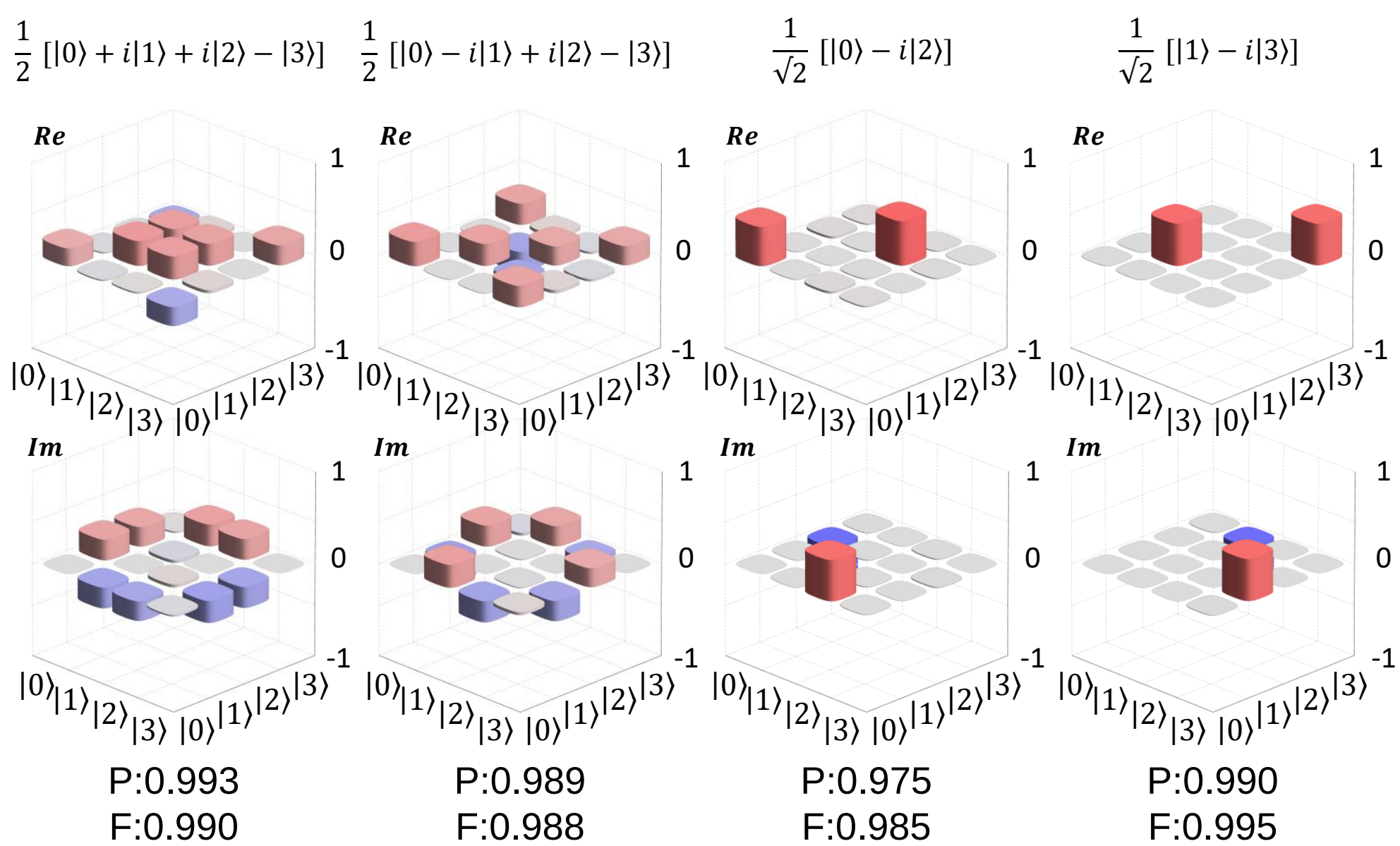
$\frac{1}{2}[|0\rangle + i|1\rangle + i|2\rangle - |3\rangle]$
$\frac{1}{2}[|0\rangle - i|1\rangle + i|2\rangle - |3\rangle]$
$\frac{1}{\sqrt{2}}[|0\rangle - i|2\rangle]$
$\frac{1}{\sqrt{2}}[|1\rangle - i|3\rangle]$
Re
Im
P:0.993
F:0.990
P:0.989
F:0.988
P:0.975
F:0.985
P:0.990
F:0.995

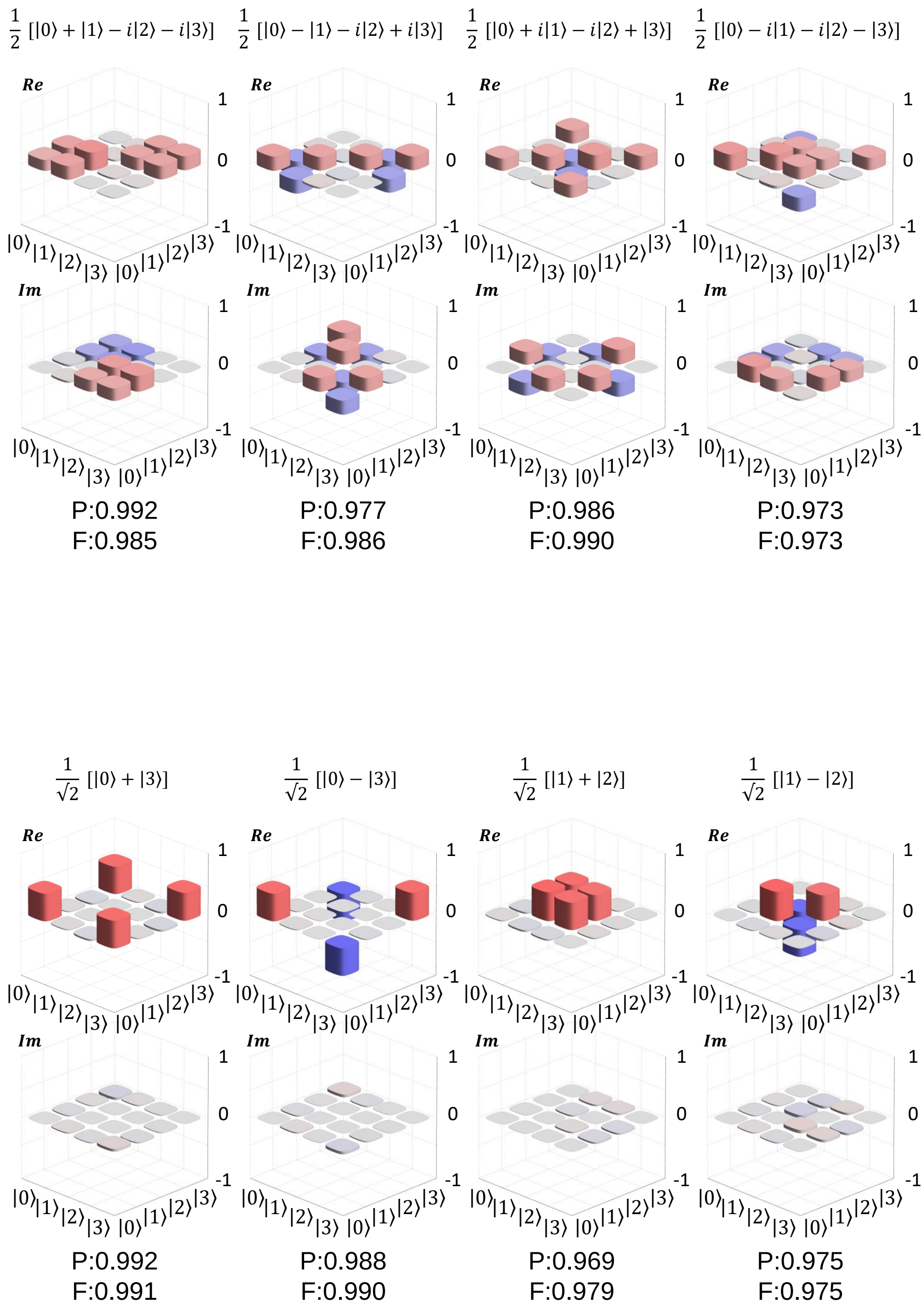

Supplementary Figure S10 | Quantum state tomography on the PPLNOI ququart chip using the integrated PPLNOI source. Each panel shows reconstructed density matrices with fidelity F and purity P. Mean values over 40 prepared states are $P = 0.9829$ and $F = 0.9859$.

Using the same sensitivity check as in Supplementary Note 5, we also evaluated the PPLNOI-source VQE data by comparing the COBYQA result with averages over the lowest $K = 5, 10$, and 15 measured energy points at each interatomic distance. This analysis checks how the chemical-accuracy assessment for the PPLNOI measurements depends on the number of lowest-energy points averaged, as shown in Supplementary Figure S11.

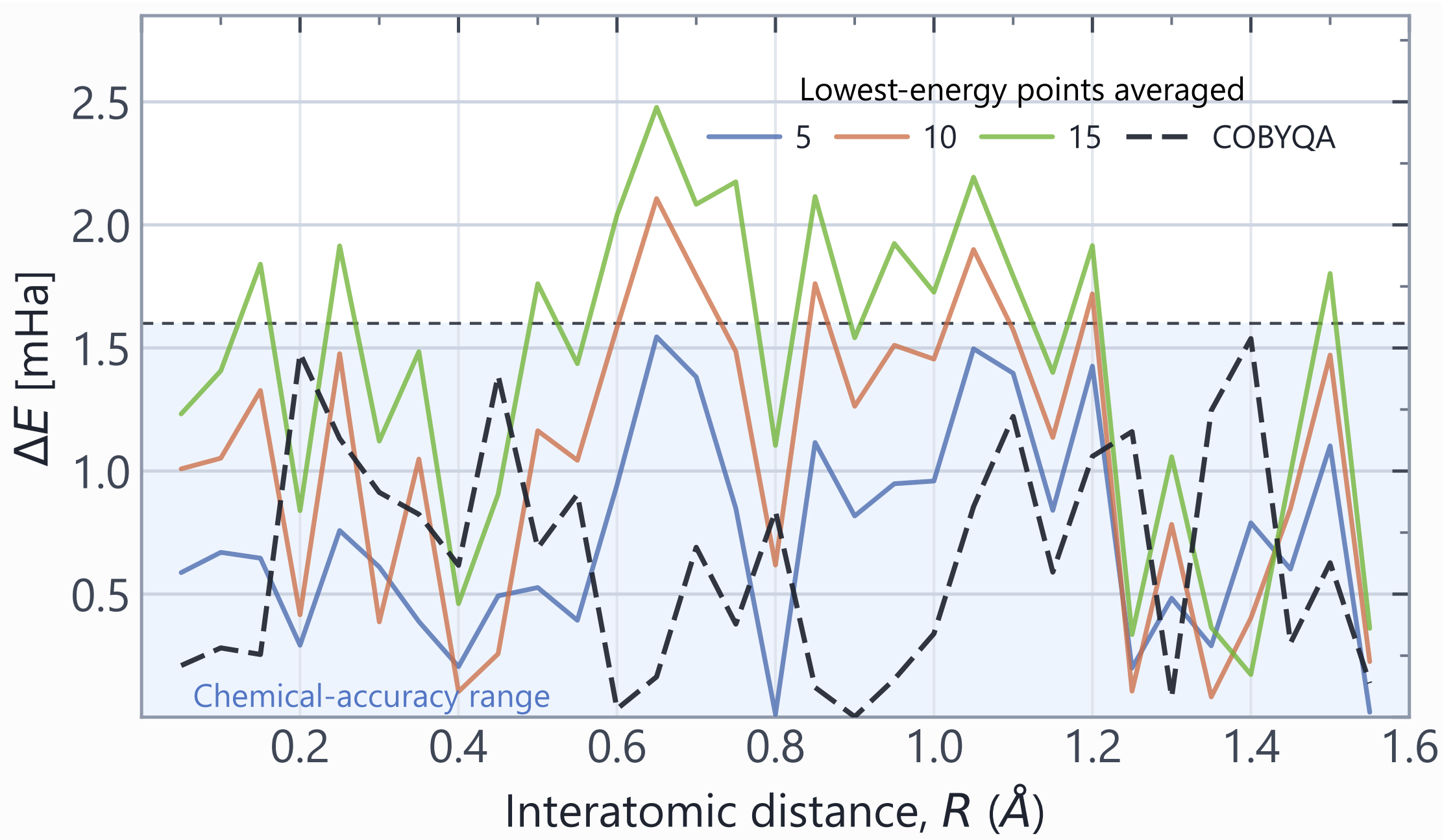

Supplementary Figure S11 | Sensitivity of the chemical-accuracy assessment to the number of lowest-energy points averaged for the PPLNOI-source VQE measurements. The curves show the absolute deviation ΔE from theory for the COBYQA result and for averages over the lowest $K = 5, 10$, and 15 measured energy points at each interatomic distance. The shaded region indicates the 1.6 mHa chemical-accuracy range.
